# CRISPR-Based Multiplex Detection of Human Papillomaviruses for One-Pot Point-of-Care Diagnostics

**DOI:** 10.1021/acssynbio.3c00655

**Published:** 2024-02-13

**Authors:** Ahmed Ghouneimy, Zahir Ali, Rashid Aman, Wenjun Jiang, Mustapha Aouida, Magdy Mahfouz

**Affiliations:** † Laboratory for Genome Engineering and Synthetic Biology, Division of Biological Sciences, 4700 King Abdullah University of Science and Technology, Thuwal 23955-6900, Saudi Arabia; ‡ Division of Biological and Biomedical Sciences, College of Health and Life Sciences, Hamad Bin Khalifa University, Education City, Qatar Foundation, P.O. Box: 34110 Doha, Qatar

**Keywords:** CRISPR-Cas, human papillomavirus, sexually transmitted infections, molecular diagnostics, multiplex detection, point-of-care detection, cervical cancer

## Abstract

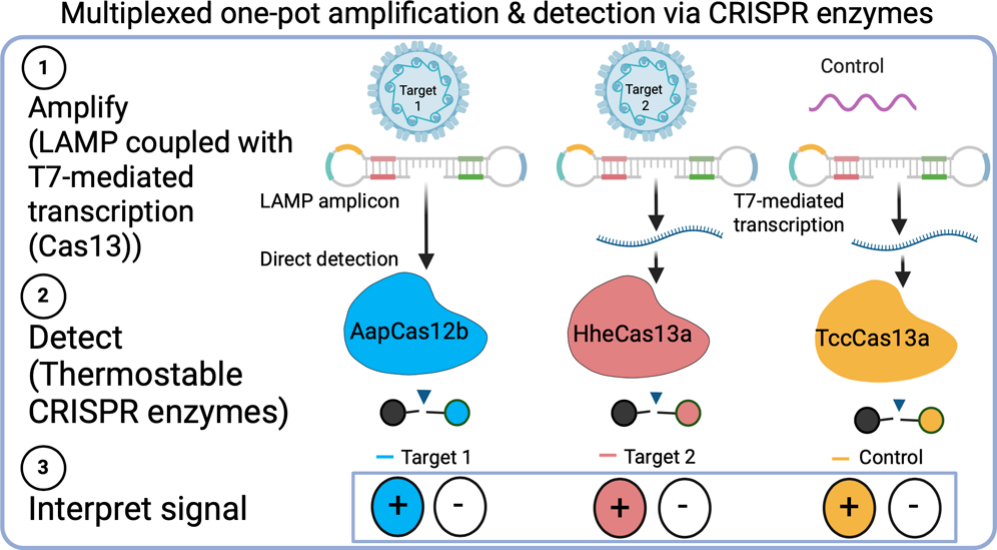

The World Health Organization’s global initiative toward eliminating high-risk Human Papillomavirus (hrHPV)-related cancers recommends DNA testing over visual inspection in all settings for primary cancer screening and HPV eradication by 2100. However, multiple hrHPV types cause different types of cancers, and there is a pressing need for an easy-to-use, multiplex point-of-care diagnostic platform for detecting different hrHPV types. Recently, CRISPR-Cas systems have been repurposed for point-of-care detection. Here, we established a CRISPR-Cas multiplexed diagnostic assay (CRISPRD) to detect cervical cancer-causing hrHPVs in one reaction (one-pot assay). We harnessed the compatibility of thermostable AapCas12b, TccCas13a, and HheCas13a nucleases with isothermal amplification and successfully detected HPV16 and HPV18, along with an internal control in a single-pot assay with a limit of detection of 10 copies and 100% specificity. This platform offers a rapid and practical solution for the multiplex detection of hrHPVs, which may facilitate large-scale hrHPV point-of-care screening. Furthermore, the CRISPRD platform programmability enables it to be adapted for the multiplex detection of any two nucleic acid biomarkers as well as internal control.

## Introduction

Globally, human papillomavirus (HPV) infections contribute approximately 4.5% of all malignancies, accounting for 8.6% of cancer cases in women (the third most prevalent cause with a high mortality) and 0.8% of cases in men.^1−3^ In 2020, nearly 600,000 women were diagnosed with cervical cancer worldwide, leading to 300,000 associated deaths.^4^ Based on the severity of this preventable cancer, the World Health Organization (WHO) has issued a global 90–70–90 call-to-action, setting clear targets to reduce cervical cancer by 2030 and irradicate HPV by 2100 through increasing HPV vaccination to 90% of the world’s population, implementing twice-lifetime screening in 70% of the female population, and treating 90% of cervical cancer patients.^5^ In developed countries, government-funded cancer screening and HPV vaccination programs have reduced the incidence and mortality of cervical cancer. However, in less-developed countries, cervical cancer remains a leading cause of cancer-related deaths.^6^

HPV infection is transmitted through sexual contact, and it is estimated that 80% of the sexually active population will experience at least one infection with HPV in their life span. Persistent infection by high-risk HPV (hrHPV) strain(s) and expression of HPV oncogenes leads to cervical intraepithelial neoplasia (CIN). Cervical disease is classified into three stages: CIN1 represents the transient infection by HPV and has a high probability (90%) of HPV regression and clearance. CIN2 is the next stage characterized by persistent replication but unproductive infection in the basal layer. CIN3 is the high-grade precancerous stage, and if not regressed, epithelial-layer malignancy leads to cervical cancer.^7−9^ The progressive nature of HPV infection and CIN provides an HPV-related cancer-prevention window as long as HPV is detected early and cervical disease is intercepted in the initial stages (CIN1 or CIN2).

The replicative genome of HPV consists of a double-stranded circular DNA of approximately 7900 base pairs. The genome contains eight overlapping open reading frames: six early (E) genes (E1, E2, E4, E5, E6, and E7) and two late (L) genes (L1 and L2). The E1 and E2 genes encode proteins involved in regulating HPV replication and transcription of early proteins, and the E6 and E7 genes encode oncoproteins.^10^ The L1 and L2 genes encode the major and minor capsid proteins, respectively. Persistent hrHPV infection leads to the constitutive expression of E6 and E7, which are responsible for host cell cycle regulation via controlling p53, pRB, and DNA repair inhibition, ultimately establishing cancer hallmarks and tumorigenesis at the upper cervix cell layer.^11^ The two most oncogenic hrHPV genotypes are HPV16 and HPV18, correlated with 95% of HPV-positive cervical cancer cases.^12^ Early stages of hrHPV-associated cervical cancer are asymptomatic, making HPV testing with subtyping for HPV16 and HPV18 E6 and E7 targets a valuable primary screening tool with higher-throughput, sensitivity, and reproducibility than standard cytology tests.^13^

Screening for cervical cancer through cytology, specifically the Pap test, is an old and prevalent technique for cancer screening, especially within organized screening programs. Developed countries have witnessed a notable reduction in cervical cancer incidence and mortality as a result of this technique.^14^ Despite being hailed as one of the most successful disease prevention initiatives, its effectiveness is notably constrained in developing regions. The cytology-based screening method mandates well-established infrastructure, repeated testing, and visits for the identification of women requiring treatment, involving not only a cytopathologist but also a colposcopy specialist and a pathologist. Essential for the success of such screening programs are training and continuous awareness.^15^ Nevertheless, the application of the Pap smear has not yielded comparable results in developing areas, likely due to the intricate and costly nature of the entire process. Moreover, the current Pap test fails to detect approximately 50% of high-grade precursor lesions and cancers during a single screening.^16^

Early detection of hrHPVs and associated malignancies is crucial for guiding effective preventative strategies against hrHPV-related cancers. Large-scale self-testing programs can substantially limit the spread of the virus, reduce infection rates, and facilitate the early prognosis of cancer development. However, the current gold standard method (qPCR) is confined to centralized laboratory settings, which require trained personnel, and is not feasible for in-home self-testing or mass screening in resource-constrained and conservative societies. Therefore, there is an urgent need for simple, accurate, specific, and user-friendly detection platforms to enable the early diagnosis of HPV and related cancers at home.

Recently, clustered regularly interspaced short palindromic repeats (CRISPR)/CRISPR-associated nuclease (Cas) systems have been demonstrated to be sensitive, specific, and programmable for point-of-care diagnostics of infectious viruses. The CRISPR/Cas system, originally designed for genome editing, comprises two main components: a guide RNA (gRNA) and a Cas nuclease, forming a ribonucleoprotein (RNP). The gRNA identifies a complementary target, and the Cas nuclease cleaves that target (*cis*-cleavage).^17,18^ However, Cas12 and Cas13 nucleases exhibit the collateral cleavage (*trans*-cleavage) of bystander DNA and RNA molecules.^19,20^ The collateral cleavage feature of Cas12 and Cas13 proteins was successfully combined with quenched fluorophore DNA or RNA reporters: upon recognition of the target pathogen DNA or RNA sequence, the activated Cas effector degrades the reporter and releases the fluorophore. The collateral cleavage activity of the Cas effector was initially applied to precisely detect the Zika virus. The sensitivity of the CRISPR-Cas diagnostic platform was enhanced through target preamplification via isothermal amplification through Recombinase Polymerase Amplification (RPA) or Loop-Mediated Isothermal Amplification (LAMP). Multiple CRISPR-Cas system modalities using RPA or LAMP, known as SHERLOCK, SHERLOCK-like, HOLMES, HOLMESv2, and ISCAN, achieved clinically relevant sensitivity and specificity.^20−23^ CRISPR-based diagnostics were comprehensively reviewed previously.^24^ In these systems, nucleic acids are preamplified via polymerase chain reaction (PCR) or isothermal amplification.^24^ PCR boasts high specificity and sensitivity but is centralized, requiring costly equipment, trained personnel, and long turnaround times.^25^ Isothermal amplification methods offer a decentralized, cost-effective alternative but may amplify nontarget nucleic acids.^26,27^

Combining the sensitivity of isothermal amplification and the specificity of CRISPR/Cas holds the potential to establish a new decentralized, gold standard point-of-care diagnostic test. Such platforms potentially meet the WHO ASSURED (affordable, sensitive, specific, user-friendly, rapid, equipment-free, delivered)^28^ guidelines for point-of-care diagnostics development. However, the existing CRISPR-based detection platforms are not suitable for multiplex detection of hrHPVs in a one-pot assay, primarily due to reaction chemistry incompatibility and nonspecific cross-collateral cleavage activity of the reporter molecules by different Cas effectors, necessitating separate reactions for different targets or separate steps for amplification and detection. SHERLOCKv2 detected 4 targets with one Cas12 and three Cas13 effectors, but the reaction required two separate steps: one for amplification and another step for detection.^29^ Tian et al. developed a multiplex reaction with Cas12 and Cas13 for the detection of two targets, but was also limited to two separate steps, amplification and detection.^30^ Similarly, multiple-target detection via LAMP-based amplification has posed challenges related to specificity.^31−33^ To overcome these issues and create a highly sensitive and specific detection system, we previously coupled LAMP amplification with CRISPR detection in a one-pot setup for infectious virus nucleic acid detection.^34^ However, detecting more than two targets remained challenging due to the lack of thermostable Cas effectors compatible with LAMP. Recent developments, including the discovery of thermostable Cas13 effectors like TccCas13a^34^ and HheCas13a,^35^ along with multiple thermostable Cas12 effectors like AapCas12b^23^ and BrCas12b,^36^ have expanded the multiplex detection possibilities. One-pot multiplexed detection of cancer-causing hrHPVs would enable early treatment of cervical cancers, intervention strategies, and policy development, potentially decreasing the toll of cervical cancer.

Here, we designed, built, and tested the CRISPRD platform, a novel approach capable of simultaneously and sensitively detecting three independent nucleic acid targets in a one-step, one-pot reaction in less than 1 h. The CRISPRD platform employs a two-layer amplification system to maximize sensitivity (limit of detection, LoD, of 10 copies) and demonstrates high specificity for hrHPV subtypes 16 and 18, and the internal positive control RNase P. There is a critical need for hrHPV screening in both males and females, and CRISPRD seeks to fulfill this unmet need. The one-pot CRISPRD reaction can be adapted for self-testing or at-home hrHPV tests.

## Results and Discussion

### The CRISPRD Workflow

**Figure 1:**
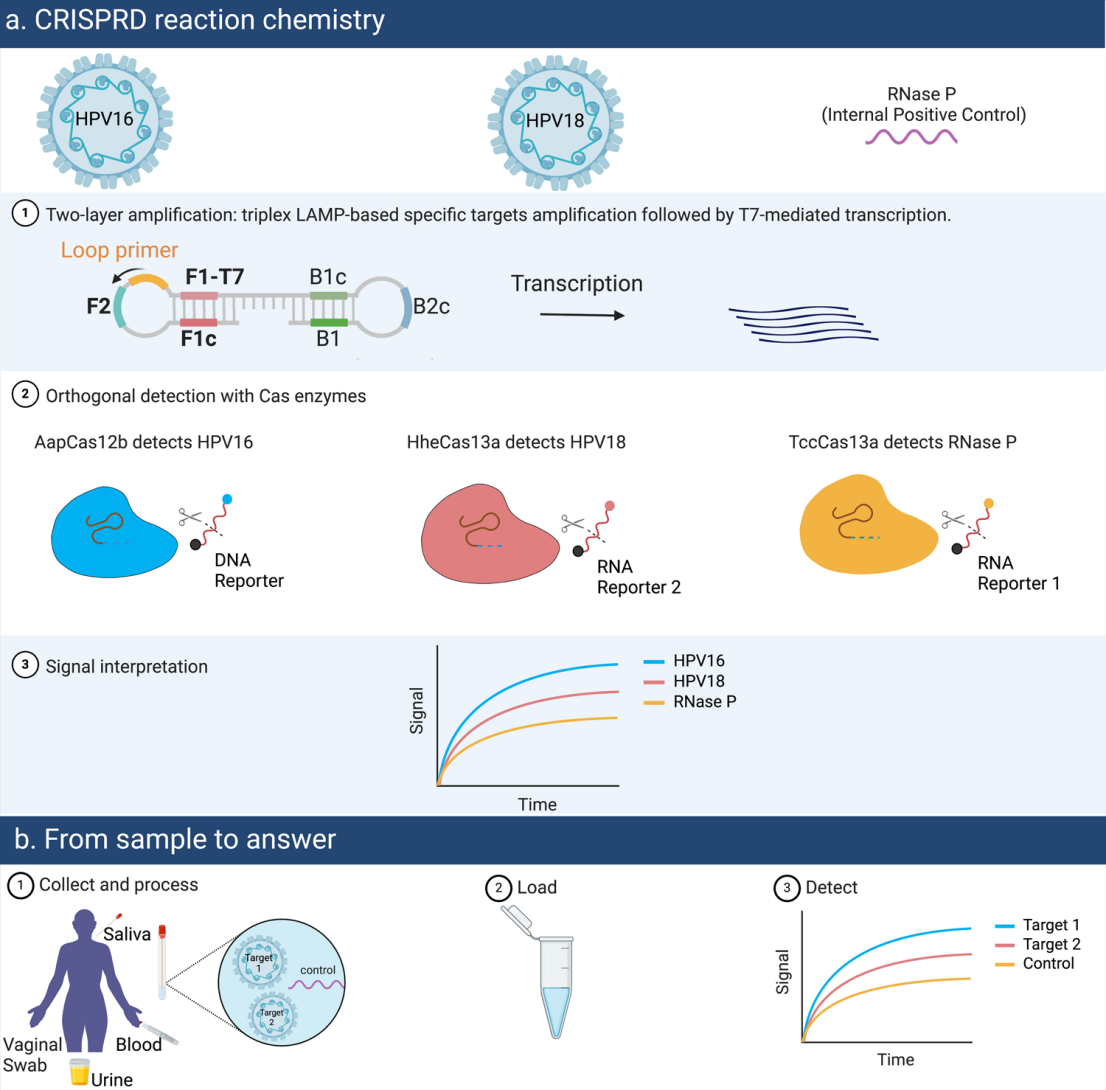
CRISPRD workflow for multiplex detection of hrHPVs. (a) CRISPRD is designed to detect three targets (HPV16, HPV16, and an internal control) simultaneously. The two layers of amplification, LAMP for DNA and T7-based transcription for RNA, are followed by CRISPR-based specific detection of the three targets in one pot. CRISPRD deploys three orthogonal Cas nucleases (TccCas13a, HheCas13a, and AapCas12b) to detect their cognate target via gRNAs and digest its associated fluorophore-conjugated DNA or RNA reporter only. Target-induced Cas variant-based digestion of the reporter releases the fluorophore as a visual fluorescent readout for pathogen detection. (b) From sample to answer: after processing the sample, nucleic acid is loaded to the one-pot CRISPRD reaction where amplification and detection occur in a single step.

We aimed to create a user-friendly one-pot multiplex diagnostic assay (CRISPR)-based multiplexed diagnostic test (CRISPRD) for the rapid mass screening of hrHPV subtypes. CRISPRD uses multiple layers of nucleic acid isothermal amplification to achieve high-level sensitivity and three different thermostable Cas effectors for specifically detecting three different targets. The initial step in the CRISPRD workflow involves a LAMP reaction to amplify the target DNAs at a constant temperature. The amplicons generated by LAMP are used as input for the subsequent detection either directly by Cas12b or indirectly by Cas13 variants (detecting RNA transcripts—all in one pot) (Figure 1).

We previously developed a one-pot reaction chemistry that couples LAMP amplification, T7-based transcription, and Cas13-based detection to efficiently identify infectious pathogens.^34^ T7-based transcription was introduced in this modality to make RNA for Cas13-based detection. Here, we aimed to optimize the one-pot detection chemistry for multiplex detection. We designed several specific primer sets with PrimerExplorer V5 (https://primerexplorer.jp/e/) to specifically amplify three targets: HPV16, HPV18, and the human internal control (RNase P). To ensure efficient RNA transcription, we introduced a T7 promoter in the LAMP primers (specifically, the Forward Inner Primer (FIP)). The amplified target DNA activates Cas12b and initiates the promiscuous cleavage of DNA reporters, whereas the RNA resulting from transcription of the target activates Cas13 and initiates the promiscuous cleavage of RNA reporters. In both cases, reporter cleavage releases a distinct fluorophore, and measurement of the resulting fluorescent signal is used for target detection. Next, we detected individual targets in single-pot reactions to ensure that the operating temperature and the reaction buffer were compatible with all enzymes.

For successful multiplexing detection, all system components must be orthogonal (reacting only with a cognate partner with no interference with other components). For that reason, orthogonality was assessed on three metrics: (1) reporter cleavage preference, i.e., finding individual Cas effectors that cut only one distinct reporter; (2) orthogonal fluorescence with the goal of choosing fluorophores with distinct excitation–emission spectra; and (3) orthogonal gRNA/Cas specificity with the goal of finding Cas effectors activated by their distinct gRNA only.

For multiplex diagnostics, the reporter systems must be orthogonal, displaying a unique signal for each target. We therefore aimed to select orthogonal Cas effectors to recognize different reporters and orthogonal fluorophores with different excitation–emission spectra. We chose two Cas13a effectors (TccCas13a and HheCas13a), each preferring a specific RNA reporter for *trans* cleavage (TccCas13a recognizing rArG or Mix RNA reporters while HheCas13a recognizes Poly-U reporters)^34,35^ and AapCas12b, which is widely known to cut single-stranded DNA (ssDNA) reporters. We designed three reporters, each with different fluorophores: FAM, HEX, and ROX which have different excitation–emission peaks to enable the detection of the three independent targets in an orthogonal fashion.

Importantly, the entire CRISPRD platform operates within a closed-lid, single-pot reaction to prevent cross-contamination. Additionally, the two steps, nucleic acid (DNA and RNA) amplification and gRNA–Cas effector-based detection, operate at a single temperature, eliminating the requirement for a thermal cycler and ensuring the feasibility of CRISPRD for point-of-care use. Our hypothesized triplex platform is suited for detecting cervical cancer-causing HPV16 and HPV18 along with an internal control in a one-pot reaction at a single temperature.

### Thermophilic Cas Effectors Successfully Detected Individual Targets

Although CRISPR-based diagnostics emerged as a promising point-of-care diagnostic system, it faces limitations in simultaneously detecting multiple targets because of the lack of orthogonality among different Cas effectors and cross-degradation of the reporters by the multiple Cas effectors used. Importantly, the Cas effectors should operate in a temperature range similar to the isothermal amplification method deployed to enable a single-step closed-lid reaction. Since LAMP is a very sensitive isothermal amplification method and requires a single enzyme (Bst DNA polymerase), and since we previously managed to create a one-pot CRISPR-based system that proved sensitive in detecting SARS-CoV-2,^37^ we wanted to expand the diagnostic capability of LAMP-based modalities. The challenge is to identify orthogonal thermostable Cas effectors since the number of thermostable Cas effectors is limited.

**Figure 2:**
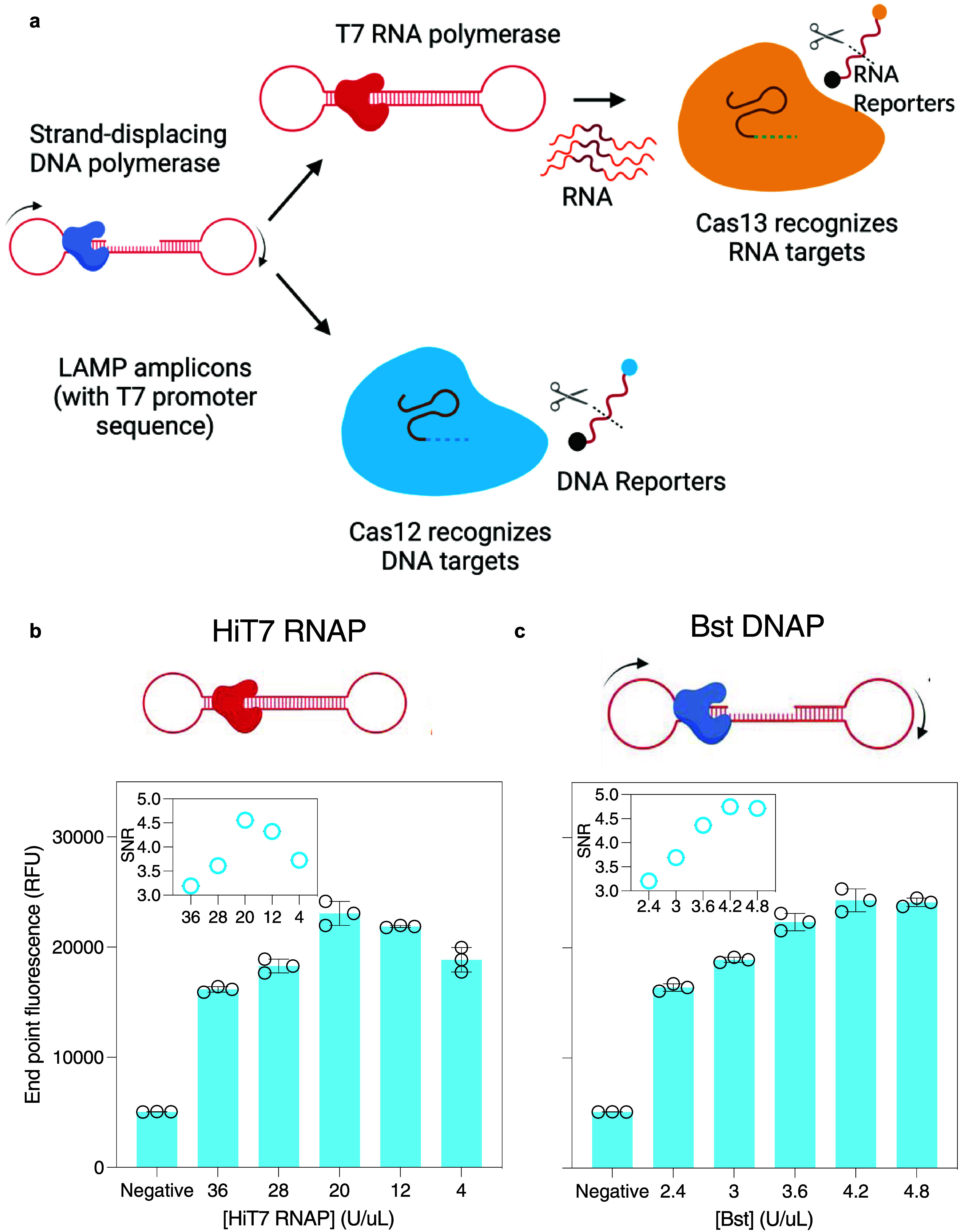
Optimization of CRISPRD single-target reaction chemistry. (a) Schematic of single target detection. The target is amplified by LAMP and either detected directly by Cas12b or transcribed to RNA then detected by TccCas13a or HheCas13a. (e, f) Optimizing the CRISPRD detection chemistry by optimizing the concentration of HiT7 RNA polymerase (RNAP) (e) and Bst DNA polymerase (f). Testing was done with TccCas13a on the RNase P target (4 ng/reaction). The signal-to-noise ratio (SNR), calculated by dividing the signal over the background (reactions lacking a target), is displayed in the inset in both figures. NTC: no template control, RNP: Ribonucleoprotein, one cycle = 2 min. The unlabeled *y*-axis follows the labeling of the *y*-axis in the left figure. All reactions were done in triplicate, and mean and standard deviation were plotted.

The emergence of thermostable Cas effectors may increase the multiplexing potential of CRISPR-Dx. For example, HheCas13a recognizes RNA targets and preferentially digests poly-U-containing RNA reporters, while recently discovered thermostable TccCas13a recognizes RNA targets and preferentially cuts rArG-residue or RNA Mix reporter (rUrG rArCrG rU)^34,35^ (Figure S7c,d). Based on the fact that Cas12 cuts DNA not RNA, we hypothesized that thermostable Cas effectors TccCas13, HheCas13a, and AapCas12b could be efficiently coupled with LAMP-based amplification to detect three targets in a one-pot reaction (Figure 2a).

To confirm our hypothesis, we purified thermostable Cas effectors TccCas13a, HheCas13a, and AapCas12b^34^ and tested them individually along with synthetic DNA (RNase P). We utilized the reaction chemistry we developed previously that couples LAMP amplitude with Cas13-based detection. Briefly, the reaction contains Bst DNA polymerase and 6 primers to enable LAMP amplification, HiT7 RNA polymerase to transcribe the LAMP amplicons, and TccCas13a to detect the RNA transcripts by cleaving RNA reporters. The reaction proceeds at a constant temperature (56 C) in a closed-lid single-step reaction that couples amplification and detection in one pot. We proceeded with testing the one-pot reaction chemistry with the different Cas effectors used in this study (HheCas13a, TccCas13a, and AapCas12b) As shown in Figure S1a–c, RNase P was efficiently amplified and detected by all Cas effectors. Of note, we tested the capability of the different Cas effectors to cleave RNase P since we had LAMP primer sets readily designed from the previous study.^37^ In the case of AapCas12b, we selected a 20-nucleotide region that is preceded with a PAM site (NTTN). TccCas13a and HheCas13a, on the other hand, can detect the RNA transcripts without requiring a PAM site.

Next, we hypothesized that optimizing the concentration of the nucleic acid amplification enzymes can improve the signal-to-noise ratio since more amplicons would activate more RNPs and cleave more reporters. We optimized the concentrations of Bst DNA polymerase (which amplifies DNA with LAMP) and HiT7 RNA polymerase (which makes RNA from the LAMP amplicons) to improve the detection efficiency of the CRISPRD platform. Our results demonstrated that adjusting the polymerase concentration substantially improved the signal (Figure 2b,c). Our single-target detection results confirmed that CRISPRD can enable the point-of-care screening for HPV, and it offers a streamlined opportunity for multiplexed one-pot detection of the hrHPVs that cause cervical cancer.

### CRISPRD Efficiently Detected hrHPV DNA

Upon persistent infection, hrHPV integrates part of its DNA genome into the host genome; therefore, it is necessary to design a diagnostic method that detects the hrHPV-integrated sequence.^38^ Accordingly, we selected the permanently integrated region encompassing the E6 and E7 genes of HPV16 and HPV18 for CRISPRD detection.

To establish our CRISPRD system for hrHPV detection, we retrieved HPV sequences from the PAVE database which is constantly updated with HPV sequences (https://pave.niaid.nih.gov). We designed several LAMP primer sets targeting the E6/E7 genes using PrimerExplorer software (http://primerexplorer.jp/lampv5e/index.html). We then designed gRNAs targeting the 20-nucleotide region between the Loop primers (manually without preference for a particular set of nucleotides). For Cas12b gRNAs, we selected regions that display a PAM sequence of NTTN. We designed multiple gRNAs specific to the E6 and E7 amplicons produced from each primer set for HPV16 and HPV18 and screened for the best-performing primer/gRNA set for each target (Figures S2 and S3). After primers and gRNAs were screened, the primer set C′ and gRNA #99 combination were superior for detecting HPV16 (using the AapCas12b RNP), whereas the primer set F and gRNA #92 combination displayed the most optimal performance for HPV18 (using the HheCas13a RNP). To establish an internal positive control, we sought to detect the widely abundant RNase P gene in humans with TccCas13a using a previously developed primer set.^39^

**Figure 3:**
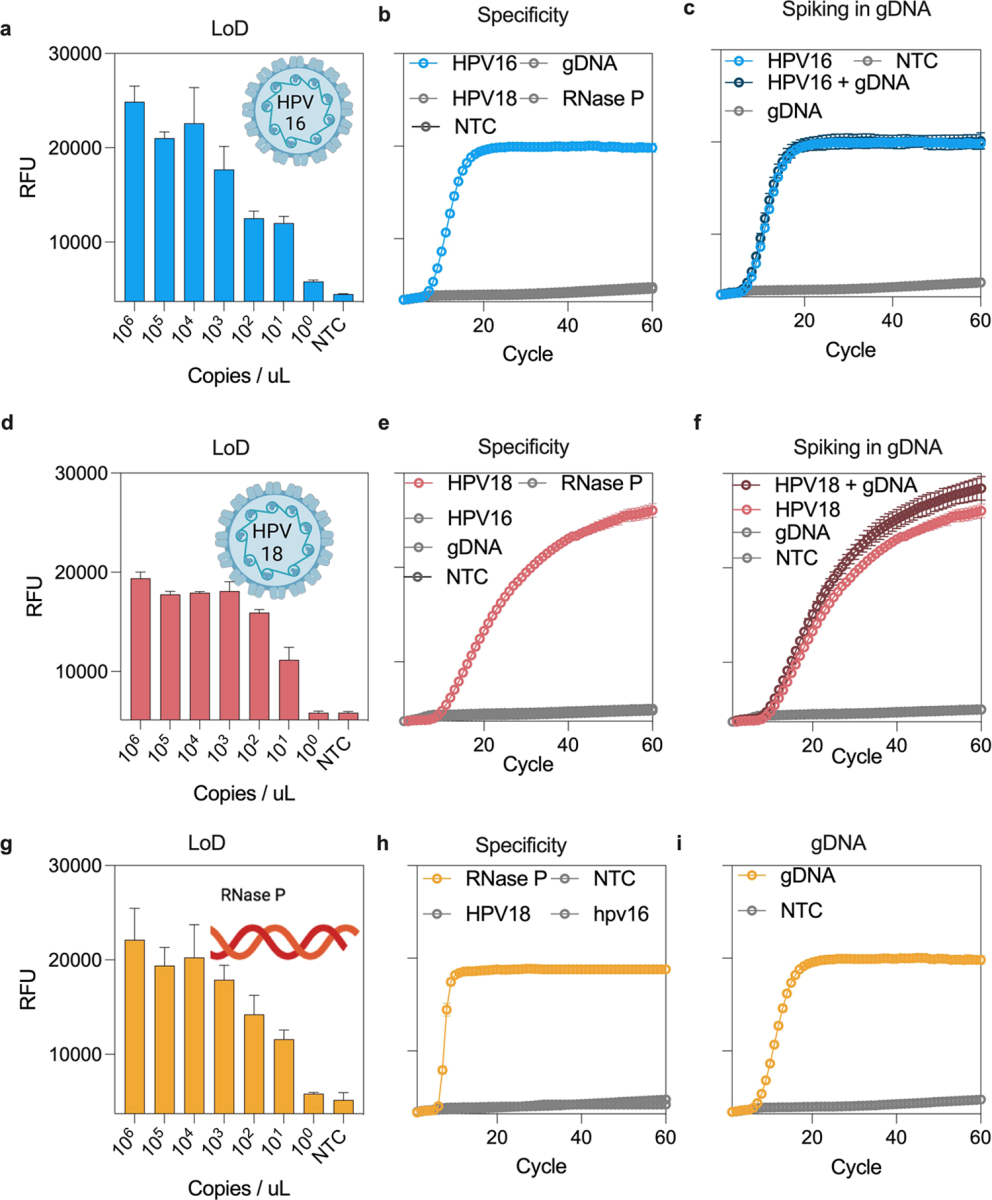
CRISPRD-based sensitive and specific detection of synthetic HPV16 and HPV18 DNA. (a) LoD for HPV16 with primer set C′ and gRNA #99 with AapCas12b. (b) Specificity of the CRISPRD reaction for HPV16 detection. The reaction mixture was incubated with 1 ng of HPV16, HPV18, gDNA, or RNase P (single target detection reactions). (c) HPV16 synthetic DNA fragments were spiked into 100 ng/μL gDNA to a final concentration of 1 ng/μL. (d) LoD for HPV18 using primer set F and gRNA #92. (e) Specificity of the CRISPRD reaction was determined for HPV18 detection. The reaction was incubated with 1 ng of HPV16, HPV18, gDNA, or RNase P (single target detection reactions). (f) HPV18 synthetic DNA was spiked into 100 ng/μL gDNA to a final concentration of 1 ng/μL. (g) LoD for RNase P with primer set Pop7 and gRNA #99 with TccCas13a. (h) Specificity of the CRISPRD reaction for RNase P detection. The reaction mixture was incubated with 1 ng of HPV16, HPV18, or RNase P (single target detection reactions). (i) Detection of RNase P in gDNA: 10 ng of gDNA was incubated in the CRISPRD reaction for RNase P detection. NTC: No-template control, RFU: relative fluorescence units, Cycle: 2 min (two-tailed Student *t* test; n.s., not significant; *P < 0.05 1;**P < 0.01; ***P < 0.001; ***P < 0.0001; All plots show mean ± SD for *n* = 3 replicates).

The CRISPRD reaction must be sensitive and specific to only HPV16 or HPV18 and should not cross-react with the human genome. We assessed the LoD for detecting HPV16 and HPV18 as well as RNase P with various dilutions of synthetic DNA, ranging from 0 to 1 million copies per μL, we found that our CRISPRD reactions could detect as low as 10 copies/μL of HPV16, HPV18, and RNase P (equal to 250 copies/reaction), comparable to Cobas HPV test from Roche^40^ (Figure 3a,d,g). Of note, in the case of HPV18, we began the study by designing four primer sets and set A was the best performing. However, our CRISPRD reactions using Set A for HPV18 could not detect below 10,000 copies/μL (Figure S4). To overcome this, we designed multiple LAMP primer sets and screened them for a better-performing primer set for detecting HPV18. Eventually, primer set F with gRNA#92 demonstrated very sensitive detection of HPV18 (Figure 3d). This indicates that robust primer screening can improve the sensitivity of a test quite significantly.

Next, we conducted a specificity test by incubating human DNA in the CRISPRD reaction targeting HPV16, HPV18, or RNase P. The test exhibited specificity toward HPV16 and HPV18, showing no interference with human genomic DNA (Figure 3b,e). Furthermore, CRISPRD successfully detected RNase P in human genomic DNA (Figure 3i). Additionally, each gRNA-Cas specifically detected its respective target without cross-reactivity with other targets (Figure 3b,e,h). Moreover, we spiked HPV16 and HPV18 into human genomic DNA and confirmed that the CRISPRD method detected both HPV16 and HPV18 precisely (Figure 3c,f,i).

### Development of a CRISPRD One-Pot Multiplexed Detection Assay

#### Orthogonality Assessment

**Figure 4:**
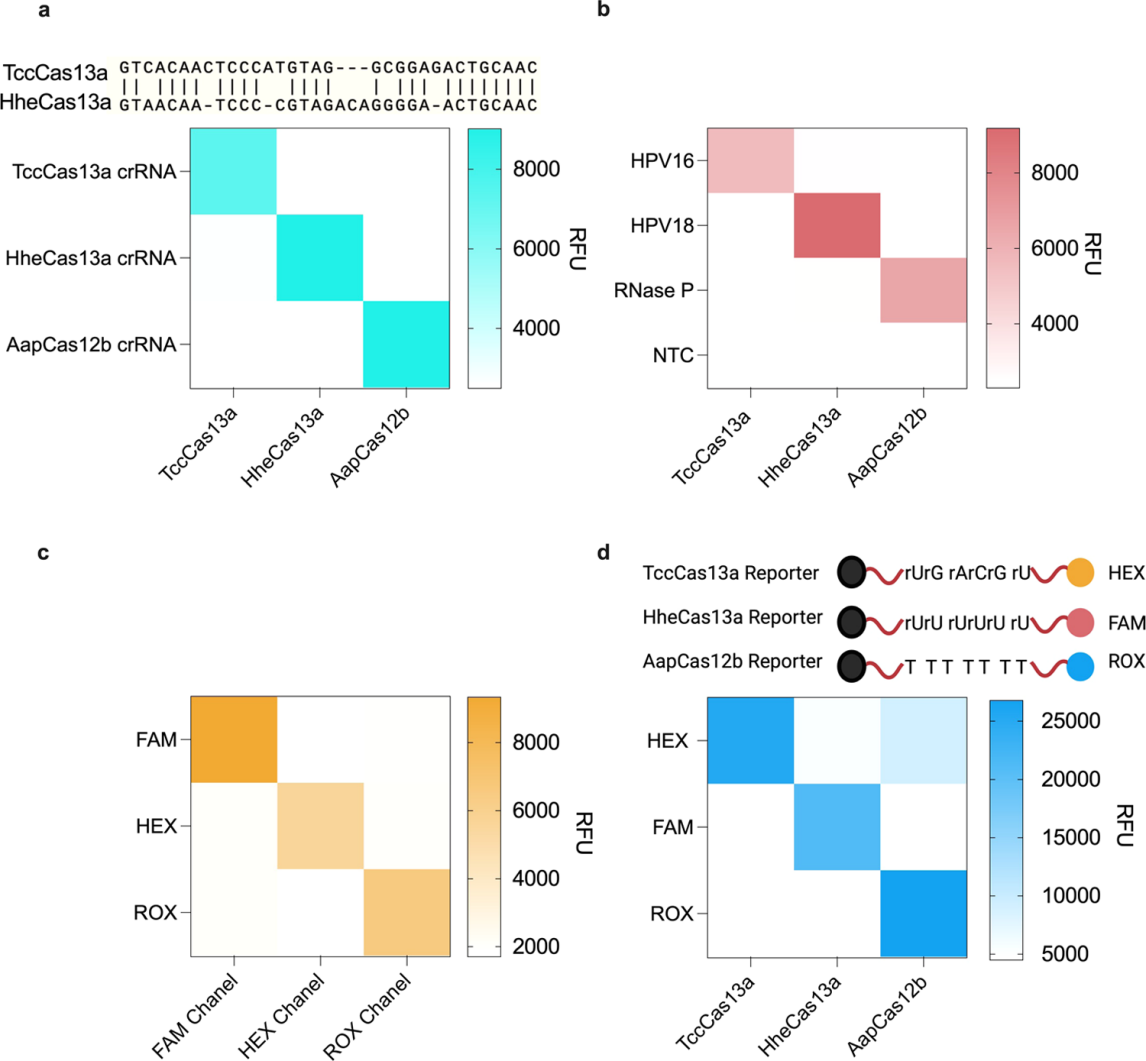
CRISPRD orthogonality. (a) Orthogonality of the CRISPR/Cas activation. Each Cas effector was tested for its ability to form an RNP with gRNAs from other effectors. (b) Orthogonality and specificity of the target. Each RNP was incubated with all three different targets to test whether targets could activate incorrect RNPs. (c) Orthogonality of the excitation and emission of fluorophores. Three different CRISPRD reactions were carried out to detect RNase P and the cleavage of reporters carrying three different fluorophores. Each reaction was read through three different channels in the qPCR machine (Bio-Rad). (d) Orthogonality of the reporter preference. Activated Cas effectors were incubated with different reporters to check if there was any aberrant cleavage of reporters. All reactions were conducted in triplicate, and the mean signal is visualized by heat maps. Panels (a–c) had 1 ng of template per reaction, while panel (d) had 4 ng of template per reaction.

Our next objective was to verify the complete orthogonality of the system in terms of (1) gRNA/Cas specificity, (2) target detection, (3) fluorescence readout, and (4) reporter cleavage preference. To confirm that the gRNAs are not interchangeable between Cas effectors, we conducted reciprocal incubations of TccCas13a with the gRNAs for HheCas13a or AapCas12b (same setup for all Cas effectors) to ensure that gRNAs would solely activate their respective Cas13 effectors without any cross-reactivity. Our findings confirmed that the gRNAs reacted specifically with their cognate Cas effector (Figure 4a). Further, we incubated each RNP and the correct cognate reporter with the different targets and confirmed that each RNP is solely activated by its respective target. To this end, we incubated the TccCas13a RNP targeting HPV16 in a CRISPRD reaction to see if it was aberrantly activated by HPV18 or RNase P. We conducted similar experiments to test the target specificity of HheCas13a and AapCas12b. Our results demonstrated that the RNPs were activated only in the presence of their specific target (Figure 4b).

Next, we focused on establishing the orthogonality of the reporter systems. We determined that HEX, FAM, and ROX offered the highest sensitivity for detection (Figure S5). We validated the orthogonality of these fluorophores by subjecting the channels of the fluorometer to all signals. The fluorophores exhibited distinct emissions spectra. Specifically, the FAM channel exclusively detected the FAM reporter, the HEX channel exclusively detected the HEX reporter, and the ROX channel exclusively detected the ROX reporter (Figure 4c).

Next, we incubated activated RNPs from each of the three effectors with each reporter to confirm that each Cas effector would only cleave the corresponding reporter and not interfere with the other reporters in the reaction. We found that both of the Cas13 effectors cleaved only their corresponding reporter molecule (Figure 4d). Nonetheless, TccCas13a exhibited slightly lower activity toward the Poly-U RNA reporter, but this effect was diminished by a decrease in the Poly-U reporter concentration (Figure S11).

Initially, we did not plan to test the activity of AapCas12b (an RNA-guided DNA nuclease) on RNA reporters since it is widely established to cut only ssDNA by collateral cleavage. However, when we later duplexed TccCas13a and AapCas12b, to our surprise, we noticed a nonspecific signal and thought it was coming from contamination (Figure S6). We then ensured that the reagents were clean and retested but observed the same nonspecific signal. We assumed that AapCas12b and TccCas13a were not compatible in the duplex reactions. Recently, Dmytrenko et al. showed that Cas12a2 has collateral cleavage activity on ssDNA, dsDNA, and ssRNA.^41^ We questioned whether AapCas12b has collateral cleavage activity on RNA, causing this nonspecific signal. Indeed, when we incubated an activated AapCas12b RNP with RNA Mix reporters, a signal emerged, indicating that the reporter was cleaved (Figure S7a). We then incubated the AapCas12 RNP with different RNA reporters hoping that it might have a preference for certain RNA nucleotides, but it cleaved most RNA reporters (except Poly-U reporters used with HheCas13a) (Figures S8 and S9). Then, we repurified AapCas12b from different clones and confirmed the AapCas12b-based promiscuous degradation of RNA reporters (Figure S7b).

To overcome the issue of aberrant cleavage by AapCas12b on RNA reporters, we purified another thermostable Cas12 effector. Nguyen et al. identified BrCas12b as a thermostable Cas12b with superior activity with LAMP compared to AapCas12b.^36^ The group further engineered an enhanced version (eBrCas12b) that could operate at the optimal temperature range for LAMP (60–65 °C).^42^ We purified BrCas12b and eBrCas12b to test their activity on RNA reporters. However, similar to AapCas12b, BrCas12b and eBrCas12b also digested RNA reporters (Figure S10), confirming that Cas12b effectors might share a universal RNA trans-cleavage capability.

We wanted to utilize this dual nuclease feature of AapCas12b using DNA and RNA reporters carrying the same fluorophore. We hypothesized that this dual nuclease feature might result in two cleavage events per collateral cleavage round, boosting the signal, speed, and sensitivity. However, the emergent signal from both reporters was lower than that of ssDNA reporters alone (Figure S11).

The discovery that AapCas12b cleaves RNA might have implications for genome editing applications. The application of Cas12 in genome editing as an alternative to Cas9 was enabled by the fact that Cas12 has *trans*-cleavage activity on ssDNA, which is not abundant *in vivo*. But considering that AapCas12b has collateral cleavage activity on RNA, its applications in genome editing might be limited as editing events will be accompanied by promiscuous cleavage of bystander RNA, leading to uncontrollable outcomes.

### CRISPRD-Based Duplex Detection of hrHPV

**Figure 5:**
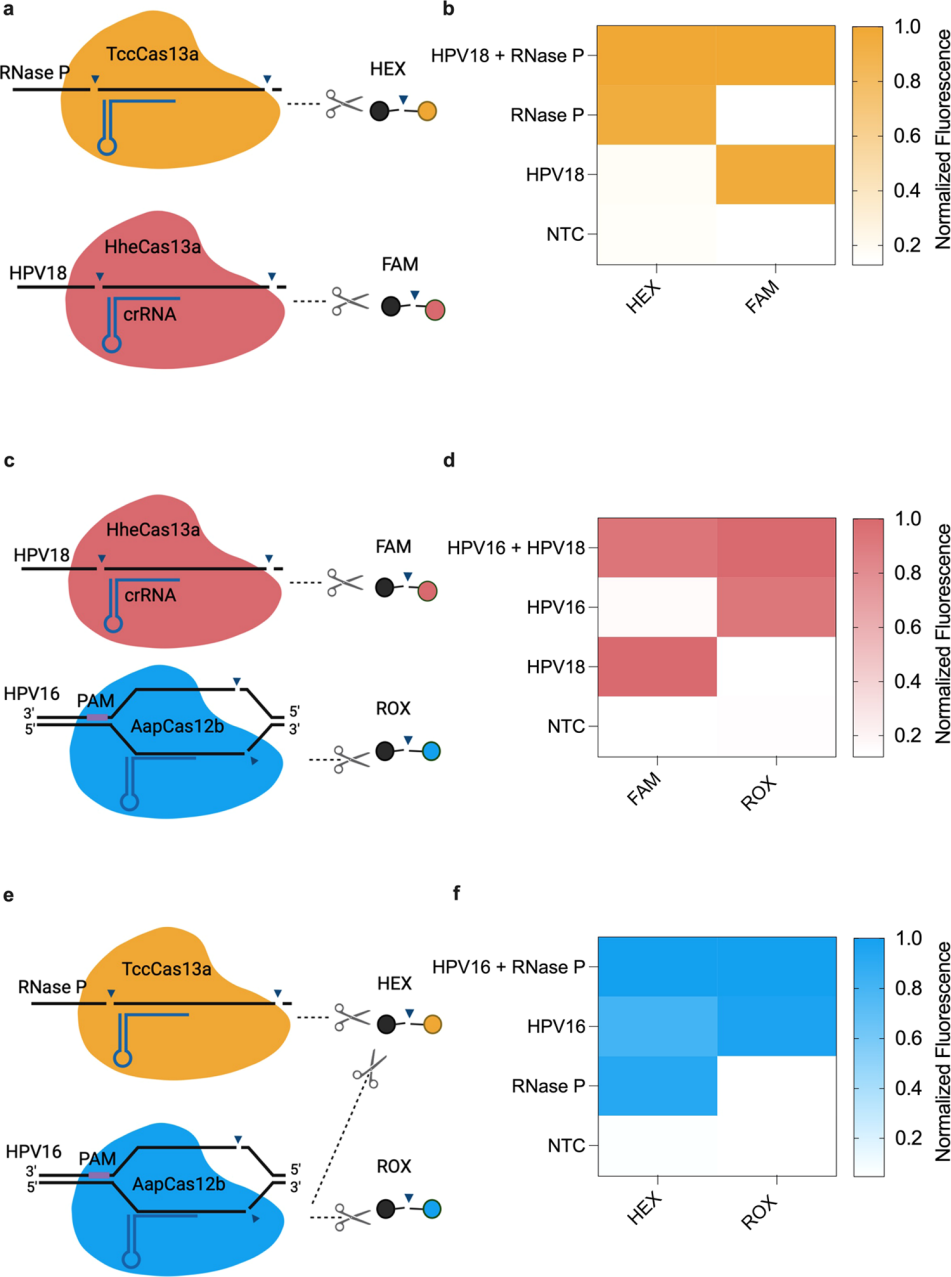
Proof of concept for duplex detection with TccCas13a, HheCas13a, and AapCas12b. (a) Schematic of the duplexed CRISPRD reaction using HheCas13a (targeting HPV18 and *trans*-cleaving FAM reporters) and TccCas13a (targeting RNase P and *trans*-cleaving HEX reporters). (b) Duplexing results in HheCas13a and TccCas13a. (c) Schematic of the duplexed CRISPRD reaction using AapCas12b (targeting HPV16 and *trans*-cleaving ROX DNA reporters) and TccCas13a (targeting RNase P and *trans*-cleaving HEX reporters) (d) Duplexing results in AapCas12b and HheCas13a. (e) Schematic of the duplexed CRISPRD reaction using TccCas13a (targeting RNase P and *trans*-cleaving HEX reporters) and AapCas12b (targeting HPV16 and *trans*-cleaving ROX reporters). (f) Duplexing results in TccCas13a and AapCas12b. Normalized fluorescence was calculated by dividing the signal over the highest signal from the corresponding reporter (*n* = 3; the mean of the normalized fluorescence is displayed on the heatmap).

The duplexed CRISPRD system included standard reagents for each CRISPRD reaction, the components for the duplexed LAMP, two assembled Cas RNPs, and two reporter molecules. We first tested the compatibility of TccCas13a and HheCas13a in our one-pot CRISPRD system (Figure 5a) and found that these two Cas enzymes were compatible in the CRISPRD duplex reaction (Figure 5b). Indeed, each effector was activated, and its respective reporter was cleaved only when the appropriate target was present. We obtained similar results with HheCas13a and AapCas12b (Figure 5c,d).

While both AapCas12b and TccCas13a possess the ability to cleave RNA Mix reporter molecules (Figure 4a), only AapCas12b can effectively cleave poly-T reporters, facilitating the identification of the activated enzyme (Figure 5e). Activation of AapCas12b results in the cleavage of both poly-T and RNA Mix reporters, resulting in ROX and HEX fluorescence. Conversely, activation of TccCas13a leads to the cleavage of only RNA Mix reporters, resulting only in HEX fluorescence. We designed TccCas13a for detecting the internal control RNase P and AapCas12b for detecting HPV16. HPV16-positive samples exhibit a signal in both the ROX and HEX channels, while negative samples display a signal only in the HEX channel. The assay is considered invalid if no signals are observed in either channel, indicating an unreliable sample.

### Development of One-Pot CRISPRD for Multiplexed HPV16, HPV18, and RNase P Detection

**Figure 6:**
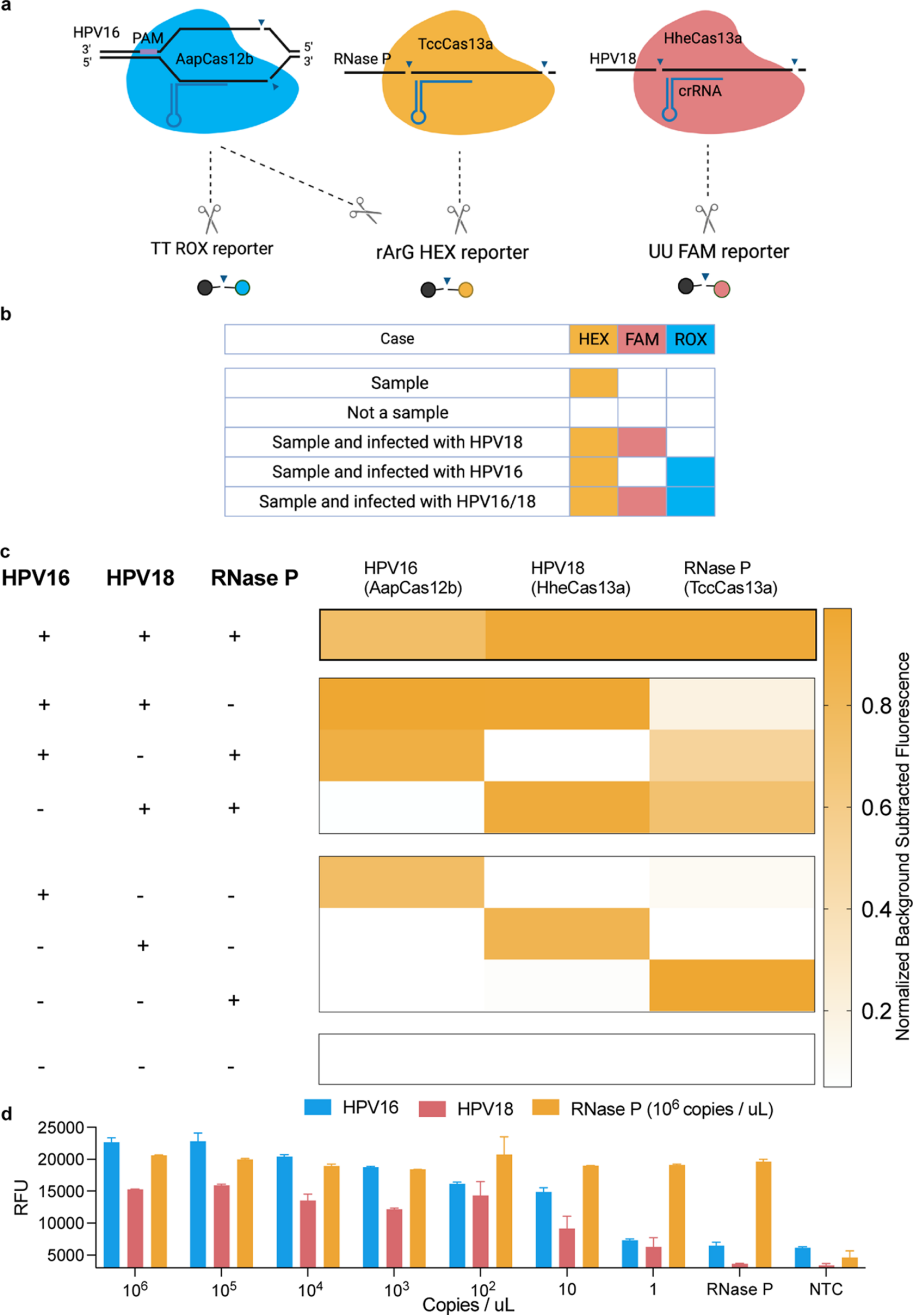
CRISPRD-based multiplex detection of HPV16, HPV18, and RNase P. (a) Schematic of the triplex CRISPRD reaction using HheCas13a (targeting HPV18 and *trans*-cleaving FAM reporters), TccCas13a (targeting RNase P and *trans*-cleaving HEX reporters), and AapCas12b (targeting HPV16 and *trans*-cleaving ROX reporters). (b) Possible outcomes of the triplex reaction, demonstrating that the cross-reactivity of AapCas12b with TccCas13a reporters will not affect the practical application. (c) Proof of concept of the triplex reaction. One master mix containing all reagents for a triplex reaction was prepared without the targets. Targets were then added in a final concentration of 1 ng/μL to each tube, and the reaction was allowed to proceed for 1 h (d) LoD of the triplex reaction. One master mix containing all triplex reagents was prepared. RNase P concentration was fixed to 1000,000 copies per microliter. The two other targets (HPV16 and HPV18) were mixed in one tube from which multiple serial dilutions were made. The reaction proceeded for 1 h; *n* = 3. Normalized background-subtracted fluorescence: background fluorescence (from tubes containing no target) was subtracted from the fluorescence signal (tubes containing targets) and then normalized to the highest signal in the corresponding reporter.

We then sought to detect three targets in a single-tube multiplexed reaction. The triplex CRISPRD reaction contained the optimized reagents in addition to three LAMP primer sets, three Cas variant RNPs, and three reporters. Given the promiscuity of AapCas12b in cutting RNA Mix reporters, we addressed this limitation by rearranging the targets (Figure 6a,b): HheCas13a targets HPV18, AapCas12b targets HPV16, and TccCas13a targets RNase P.

In practice, the third target is always a positive internal control, which indicates that the sample is intact. Accordingly, five different scenarios could result from this triplex reaction (Figure 6b): (1) If a sample contains a sufficient amount of DNA from the test subject, it must contain the internal control, in this case RNase P, which would lead to the activation of TccCas13a and the cleavage of RNA Mix HEX reporters. (2) If a sample is degraded or not enough DNA was collected, no HEX signal would emerge. (3) If the subject is infected with HPV16, this would lead to the activation of AapCas12b, which would cleave Poly-T ROX reporters and RNA Mix HEX reporters. Theoretically, the presence of both signals would indicate the presence of HPV16 in the sample but would not ensure the presence of RNase P. For diagnostic purposes, however, the presence of HPV16 is sufficient to indicate an intact sample. (4) If the subject is infected with HPV18, this would indicate it also contains RNase P, leading to the activation of HheCas13a and TccCas13a and the cleavage of Poly-U FAM and RNA Mix HEX reporters, respectively. (5) A subject that is infected with HPV16 and HPV18 would have the three signals: HEX, ROX, and FAM.

For a proof of concept, we made all combinations of targets in a one-pot triplexed CRISPRD reaction (Figure 6c). In one setup, we added all three targets to the reaction tube to ensure that tripleting is possible. Additionally, we added all two-target combinations in a triplexing reaction, and the signal that emerged was specific, except (as expected) in the combination of RNase P and HPV16 (due to the promiscuity of AapCas12b). We also added each target individually to the triplex CRISPRD reaction and detected each target in the corresponding channel for each single-target reaction.

To ensure that detecting three targets in one pot does not compromise the detection sensitivity, we assessed the LoD of each target by incubating serial dilutions of the targets in the CRISPRD reaction, attained a LoD of 10 copies/μL for all targets, and reached single-copy detection for HPV18 and RNase P (Figure 6d).

### CRISPRD Successfully Detected HPV16 and HPV18 DNA in Human Cell Lines

**Figure 7:**
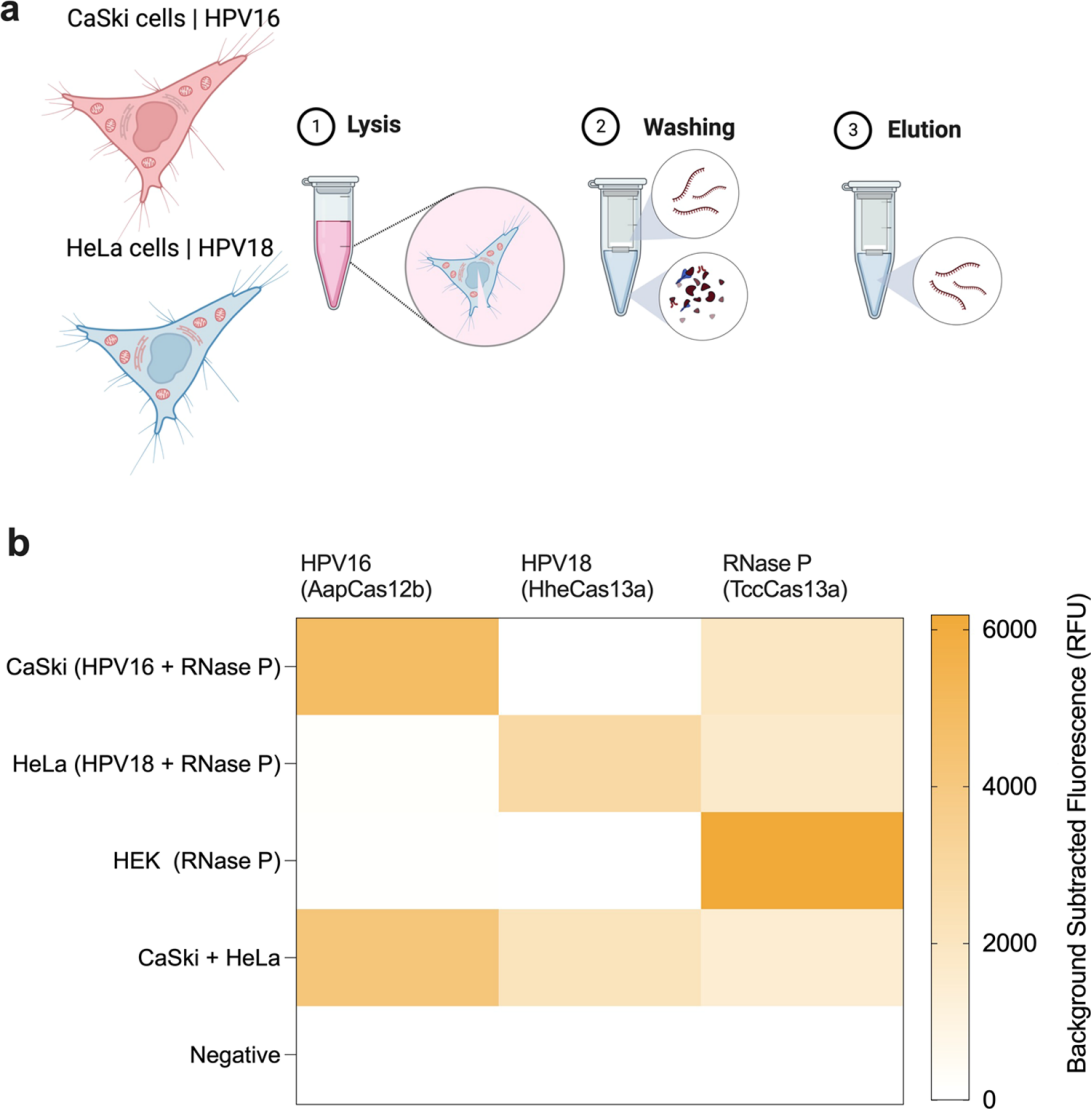
Validation of CRISPRD for detecting HPV DNA in human cell lines. (a) CaSki cells with an integrated HPV16 genome and HeLa cells with an integrated HPV18 genome were used to validate the triplex CRISPRD reaction. Human genomic DNA was extracted by a standard protocol of lysis, washing, and elution. (b) Validation of the CRISPRD reaction was performed using these cell lines. One master mix containing all reagents for a triplex reaction was prepared without adding the targets. DNA from each cell line was mixed to detect HPV16 and HPV18 targets. The reaction proceeded for 2 h (*n* = 3). HEK-293T cells (which do not contain HPV16 or HPV18 DNA) were used as negative control. The mean of the background-subtracted fluorescence is visualized in a heatmap. A total of 10 ng of total DNA extracted from the corresponding cell line was added to each reaction.

hrHPVs integrate part of their DNA genome into the host’s genome, and persistent expression of the virus oncogenes E6 and E7 leads to cervical malignancies. Human cell lines CaSki (HPV16) and HeLa (HPV18) integrated with HPV genomes were previously isolated and represent a good resource for hrHPV research.^43,44^ Thus, we designed our CRISPRD method against the hrHPV-integrated sequence to confirm the efficacy of our diagnostic module and ensure it can detect HPV DNA integrated in human DNA. Following purification of gDNA from cell lines (Figure 7a), CRISPRD detected HPV16 and HPV18 in CaSki and HeLa cells, respectively, confirming the specificity and multiplexing potential of the CRISPRD reaction chemistry (Figure 7b).

To date, most CRISPR-based platforms offer single-target detection in one pot. Multiplexing is challenging due to the nonspecific cleavage activities of the Cas effectors. Arrayed multiplexing, exemplified by CARMEN^45^ and mCARMEN,^46^ is one way to overcome the nonspecific cleavage activity of Cas effectors by performing multiple single-target detection events in distinct channels. Arrayed multiplexing enabled high-throughput multiplexing of 1000 targets in a single test.^45^ However, arrayed multiplexing suffers from several limitations, including (1) the assay is not user-friendly and not compatible with point-of-care detection, (2) requires special, separate equipment for amplification and detection, and (3) is time-consuming: results take more than a day to obtain.

Single-tube multiplexing, on the other hand, was enabled by the discovery of new Cas effectors. The fact that Cas12 nucleases cut DNA, whereas Cas13 nucleases cut RNA enabled duplex reactions. The discovery of collateral cleavage preference toward some nucleotides enabled multiplexing several Cas13 effectors, but detection was separated from amplification.^29^ Recently, SHINE.V2 reported duplex detection of SARS-CoV-2 and an internal control using Cas13 and Cas12 in one pot (with amplification via RPA).^47^

In this work, we increased the single-pot multiplexing potential by coupling LAMP with three CRISPR/Cas effectors in a single-step reaction for the detection of three targets. The CRISPRD platform can be scaled to many other diseases that require single, double, or triple biomarker detection. During the COVID-19 pandemic, there was a need for a test that differentiates COVID from influenza to contain the spread of SARS-CoV-2 and influenza. Our CRISPRD platform can be reprogrammed to detect COVID and influenza. It can also differentiate between Subtypes A and B of influenza. CRISPRD can also be applied to detect the deadly methicillin-resistant *Staphylococcus aureus* (MRSA), which requires detecting two genes.^31,48^ In general, the CRISPRD platform can detect any two nucleic acid biomarkers of interest as well as internal control in a reaction that houses LAMP-enabled amplification and CRISPR-based detection in one tube.

Although the CRISPRD platform is a step forward toward the multiplex detection of pathogens at the point of care, CRISPRD has the same limitations as most CRISPR-based detection platforms. We previously outlined the challenges and potential solutions to bring CRISPR-based detection methods to the market.^24^ Namely, the CRISPRD platform relies on a fluorimetric reading of the signal. Though colorimetric signals are preferred, especially in point-of-care setups, companies such as Egoo Health are trying to commercialize cheap fluorescence readers that can read multiple fluorometric signals at once. Additionally, the CRISPRD platform still needs to be tested with quick sample processing modalities. An ideal diagnostic kit would have a sample-to-answer either in a single tube or in multiple channels controlled by automation in small point-of-care devices.^49^ An ideal diagnostic tool should also be straightforward, inexpensive, and sensitive.^50^

## Conclusions

In this study, we screened and coupled recently discovered thermostable Cas effectors with LAMP-based amplification to create a CRISPR-based one-pot platform called CRISPRD for the multiplex detection of hrHPVs. CRISPRD enabled the first highly specific and sensitive triplex hrHPV detection method, a step toward decentralizing hrHPV diagnostics and assisting in cervical cancer mitigation according to WHO recommendations. The one-pot, single-step reaction chemistry of the CRISPRD detection module can be further developed by incorporating a quick sample preparation step and encapsulating the reaction steps in a small device to fulfill the WHO-recommended ASSURED criteria of point-of-care detection. Our CRISPRD platform offers unprecedented specificity relative to any multiplexed LAMP platform designed to date.^31−33^

## Materials and Methods

### Protein Purification

The plasmid p2CT-His-MBP-Hhe_Cas13a_WT used for HheCas13a expression was acquired from Addgene (plasmid #91871). Recombinant HheCas13a was purified according to a previously published protocol.^35^ The clone containing TccCas13a is also available in Addgene (Plasmid #199754). Expression and purification of TccCas13a followed a previously published protocol.^34^ The plasmid pAG001-His6-TwinStrep-SUMO-AapCas12b for expressing AapCas12b was obtained from Addgene (plasmid #153162), and purification followed a previously published protocol.^51^

Clones containing BrCas12b^36^ and eBrCas12b^42^ were obtained from Addgene (Plasmids #182276 and #195339, respectively). Expression and purification of BrCas12b and eBrCas12b were performed according to previously published protocols.^36,42^ After cleavage with TEV protease (from Tobacco Etch Virus), size exclusion chromatography was performed to separate BrCas12b or eBrCas12b from the MBP (Maltose Binding Protein) tag. The size exclusion buffer contained 25 mM Tris HCl (pH, 7.5), 100 mM NaCl, 10% glycerol, and 1 mM TCEP (tris(2-carboxyethyl)phosphine).

### gRNA Production

To generate the HheCas13a and TccCas13a gRNAs, 10 μM T7 promoter (oligo from IDT) was annealed to the bottom strand, which contains the repeat region and the spacer for the gRNA (single-stranded oligo). Annealing occurred in a PCR buffer with a gradual decrease of temperature from 95 to 25 °C, in 5 °C decrements every 2 min. The annealed product was in vitro transcribed (IVT) with a T7 highscribe kit (NEB) according to the manufacturer’s protocol. The RNA product was purified with zymo RNA purification kit and measured with a NanoDrop spectrophotometer.

To generate the AapCas12b, BrCas12b, and eBrCas12b gRNAs, the repeat region was used as a single-stranded DNA ultramer (IDT). To make a full-length DNA for the gRNA (containing the repeat and the spacer), the spacer was used as a reverse primer (having a shared region with the repeat region) and PCR was amplified with the forward primer T7 oligo. The PCR product was purified with Qiagen and the product was measured with a NanoDrop spectrophotometer. Then, 300–1000 ng of the PCR product was used as a template for the IVT using Hiscribe kit (NEB). The product was purified with a zymo RNA purification kit and measured with a NanoDrop spectrophotometer.

### Design and Screening of LAMP Primers

We retrieved HPV sequences from the PAVE database which is constantly updated with HPV sequences (https://pave.niaid.nih.gov). Different primer sets targeting several regions of the E6 and E7 genes of the HPV16 and HPV18 genomes were designed using PrimerExplorer v5 software (https://primerexplorer.jp/e/). Optimal primer sets that showed the best performance were determined by conducting colorimetric LAMP and CRISPRD detection assays to detect specific targets.

### Conditions for the CRISPRD Reaction (Amplification and Detection)

The reaction operates at 56C in a closed-loop single-step process that combines amplification and detection. The reaction proceeds for 1 h, and the fluorescence values are measured with qPCR machine (Biorad).

### CRISPRD Reaction for Single-, Double-, and Triple-Target Detection

The CRISPRD reaction was created in two parts: RNP assembly (incubating Cas effector with gRNA (Table 1)) followed by the addition of the assembled RNP to a tube containing the reagents in Table 2. The tables below describe the reagents used in the CRISPRD single-, double-, and triple-target detection reactions.

**Table 1:** RNP Assembly

reagents	final concentration
H_2_0	To 5 μL
isothermal buffer (NEB)	1×
gRNA	1 μM
Cas effector	1 μM

**Table 2:** Master Mix

reagents	final concentration	singlet	duplex	triplex
H_2_O	To 50 μL	+	+	+
Isothermal buffer (NEB)	1×	+	+	+
MgSO_4_ (NEB)	6 mM	+	+	+
dNTPs (NEB)	1.4 mM	+	+	+
NTPs (NEB)	0.5 mM	+	+	+
HiT7 (NEB)	20 U/μL	+	+	+
Bst DNA polymerase, exonuclease minus (Lucigen)	4.2 U/μL	+	+	+
Inorganic pyrophosphatase (NEB)	0.4 U/μL	+	+	+
primer set 1 (20×)	1×	+	+	+
primer set 2 (20×)	1×	–	+	+
primer set 3 (20×)	1×	–	–	+
FAM RNA reporter (IDT)	1 μM	+	+	+
ROX DNA reporter (IDT)	4 μM	–	+	+
Hex RNA reporter (IDT)	2 μM	–	–	+
TccCas13a RNP	80 nM	+	+	+
AapCas12b RNP	80 nM	–	+	+
HheCas13a RNP	80 nM	–	–	+
target 1	×	+	+	+
target 2	×	–	+	+
target 3	×	–	–	+

After assembling one reaction (one tube containing all reagents for amplification, detection, as well as targets), the reaction is incubated at 56 and fluorescence measurement is taken using qPCR machine (Bio-Rad).

### Testing the Collateral Cleavage Preferences of AapCas12b on ssRNA Reporters

To validate the trans-cleavage activity of AapCas12b on different reporters, we incubated 100 nm of AapCas12b, 100 nm of gRNA (gRNA CV627 targeting RNase P), 400 ng of RNase P, and 1 μM of different reporters in 1× isothermal amplification buffer (NEB) and 6 mM MgSO_4_ (NEB). The reaction proceeded at 56 °C, and real-time fluorescence was recorded for each reaction.

### Preparation of Genomic DNA from Cell Lines

We isolated nucleic acid from cell lines (HeLa, CaSki, and HEK) using the Monarch Genomic DNA Purification Kit (NEB no. T3010) following the manufacturer’s protocol.

### Copy Number Calculation for LoD Experiments

For LoD experiments, we calculated the copy number using two pieces of information:

(1) To get number of copies in 1 μg: We used the DNA Calculator https://molbiotools.com/dnacalculator.php. Simply, we first input the sequence of interest, and in the “Calculated properties” section, we get the “Number of molecules in 1 μg”.
(2) Based on the synthetic DNA specifications provided by IDT, we know the mass of the synthetic DNA fragments.

Based on information in 1 and 2, we can convert between mass and copy number.

## Supporting Information Available

CRISPRD Reaction with TccCas13a, HheCas13a, and AapCas12b (Figure S1); screening for the best-performing set of primers/crRNAs (real-time data) (Figure S2); primer screening for HPV16 and HPV18 (end point) (Figure S3); LoD using primer set A for HPV18 (Figure S4); reporter screening (Figure S5); duplexing AapCas12b with TccCas13a (Figure S6); testing the collateral cleavage activity of AapCas12b purified from two different clones (Figure S7); activity of AapCas12b on RNA reporters with different nucleotide compositions (Figure S8); reporter screening with AapCas12b real-time data (Figure S9); BrCas12b cleaves RNA and DNA reporters (Figure S10); testing AapCas12b with RNA and DNA reporters (Figure S11); activity of TccCas13a on Poly-U RNA reporters (Figure S12) (PDF)
Nucleic acid sequences used in this study (XLSX)
