# Supplementary material for: CRISPR-Based Multiplex Detection of Human Papillomaviruses for One-Pot Point-of-Care Diagnostics
Source: ACS Synth Biol. 2024 Feb 13;13(3):837–50. doi: 10.1021/acssynbio.3c00655 (PMC10949237; doi:10.1021/acssynbio.3c00655)
Supplement: Supplementary file 1 — sb3c00655_si_001.pdf [file sb3c00655_si_001.pdf]

Supplementary

CRISPR-based multiplex detection of human papillomaviruses for one-pot point-of-care diagnostics

Ahmed Ghouneimy[1], Zahir Ali[1], Rashid Aman[1], Wenjun Jiang[1], Mustapha Aouida[2] and Magdy Mahfouz[1*]

[1]Laboratory for Genome Engineering and Synthetic Biology, Division of Biological Sciences, 4700 King Abdullah University of Science and Technology, Thuwal 23955-6900, Saudi Arabia.

[2]Division of Biological and Biomedical Sciences, College of Health and Life Sciences, Hamad Bin Khalifa University, Education City, Qatar Foundation, Doha, Qatar, P.O.Box: 34110).

***Correspondence**: Magdy M. Mahfouz (magdy.mahfouz@kaust.edu.sa)

Keywords: CRISPR-Cas, Human Papillomavirus, Sexually transmitted infections, Molecular diagnostics, Multiplex detection, Point-of-Care detection, Cervical cancer

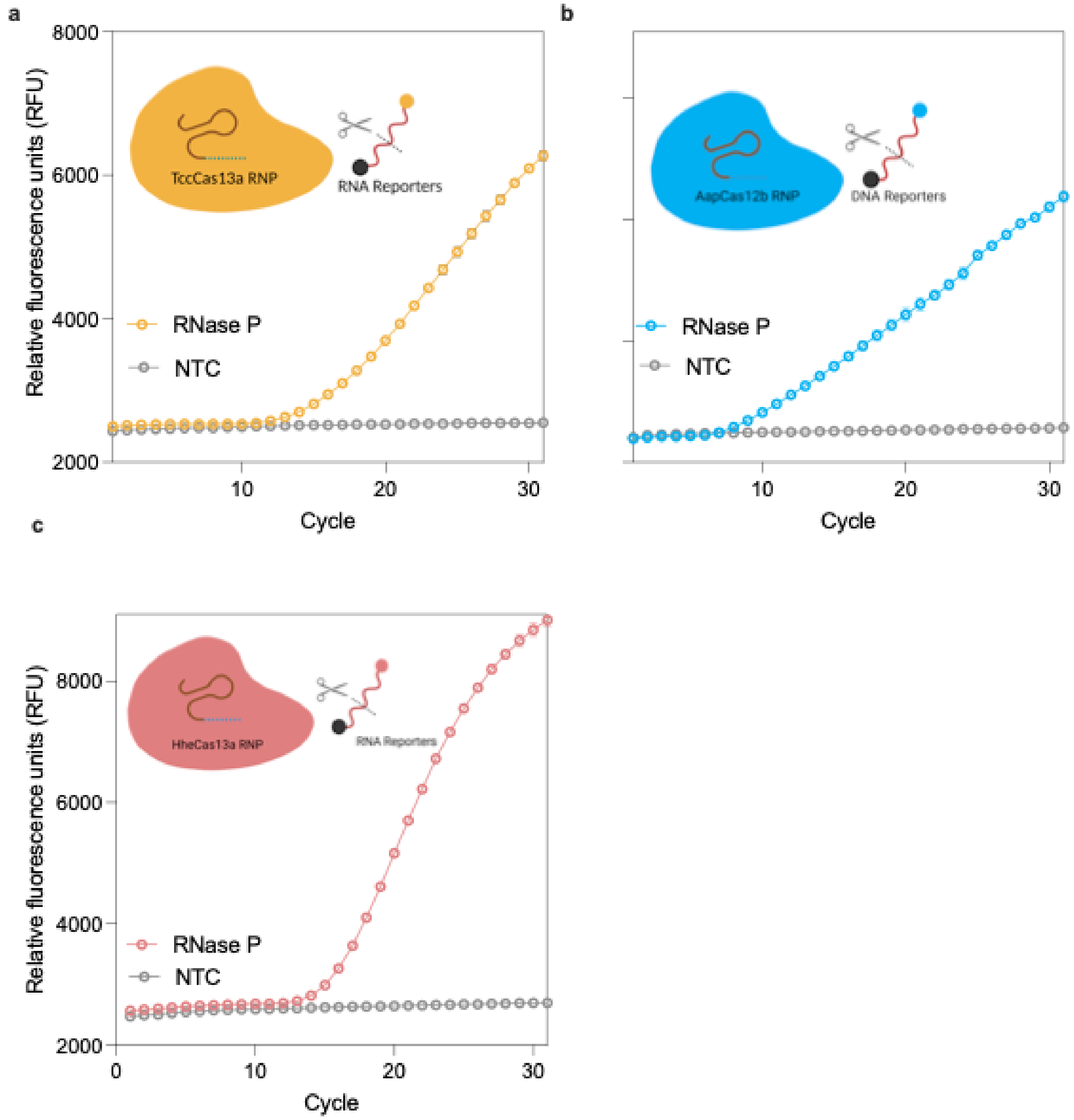

Fig. S1 | The CRISPRD Reaction with TccCas13a, HheCas13a, and AapCas12b. Testing the CRISPRD reaction for detecting synthetic RNase P DNA (1 ng/reaction) with (**a**) TccCas13a, (**b**)AapCas12b and (**c**) HheCas13a.

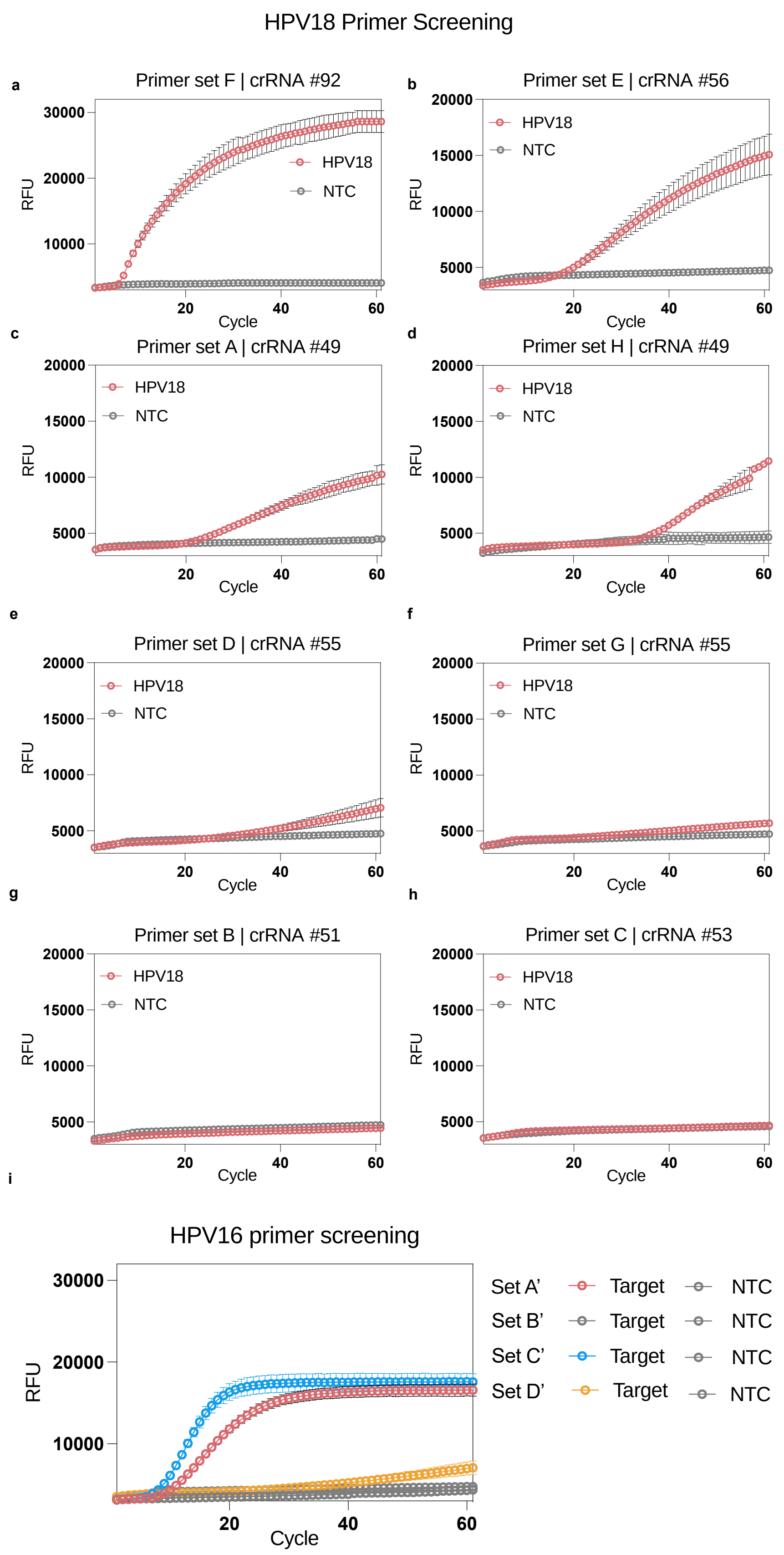
HPV18 Primer Screening
a
Primer set F | crRNA #92
b
Primer set E | crRNA #56
c
Primer set A | crRNA #49
d
Primer set H | crRNA #49
e
Primer set D | crRNA #55
f
Primer set G | crRNA #55
g
Primer set B | crRNA #51
h
Primer set C | crRNA #53
HPV18
NTC
RFU
Cycle
i
HPV16 primer screening
Set A' Target NTC
Set B' Target NTC
Set C' Target NTC
Set D' Target NTC
RFU
Cycle

Fig. S2 | Screening for the best-performing set of primers/crRNAs (real-time data) for HPV16 and HPV18. a-h Eight primer sets were screened with the CRISPRD reaction for detecting HPV18 (4 ng/reaction) with HheCas13a. **i** Four primer sets were screened with the CRISPRD reaction for detecting HPV16 (4 ng/reaction) with AapCas12b. Plotted is the mean fluorescence of n=3 technical replicates and standard deviation. crRNA and gRNA are used interchangeably in the study.

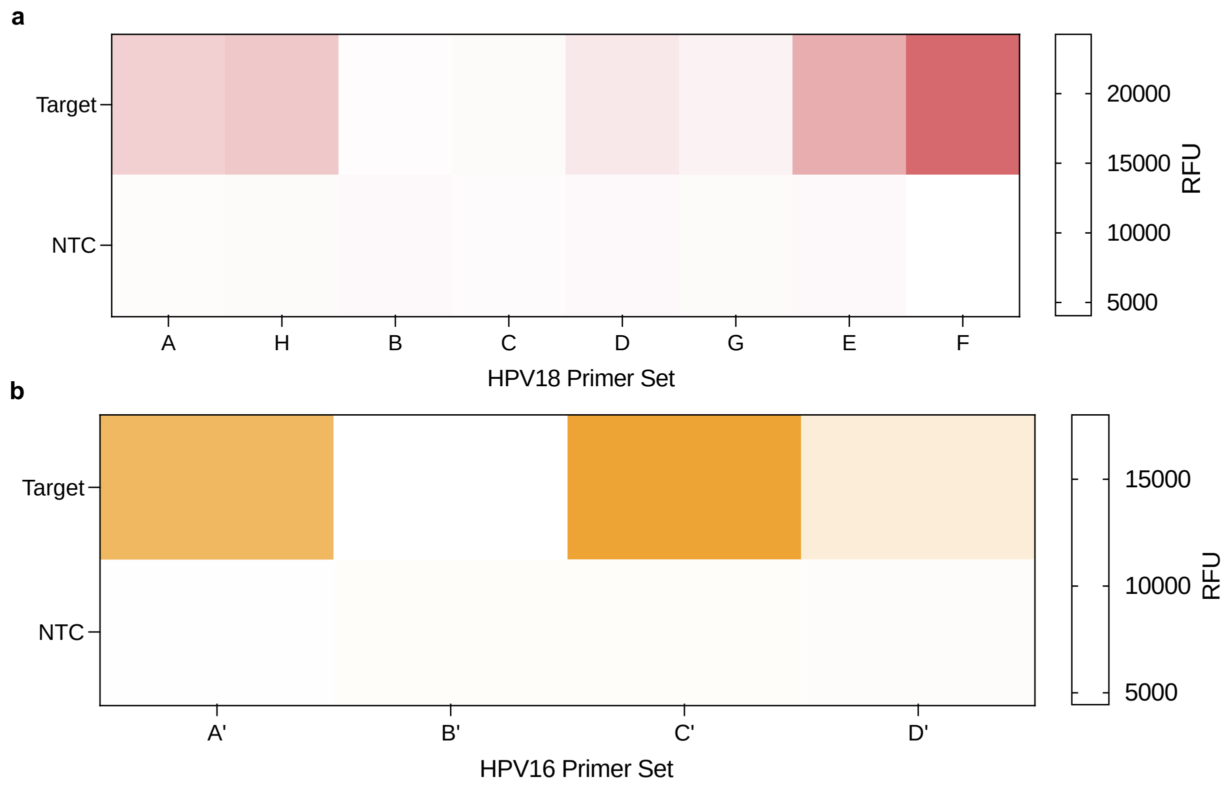

Fig. S3 | Primer screening for HPV16 and HPV18 (Endpoint). Endpoint fluorescence of a) 8 primer sets for HPV18 (4 ng/ reaction) and b) 4 primer sets for HPV16 (4 ng/ reaction) screened using the CRISPRD reaction: HheCas13a for HPV18 and AapCas12b for HPV16. Plotted is the mean endpoint fluorescence of n=3 technical replicates and standard deviation.

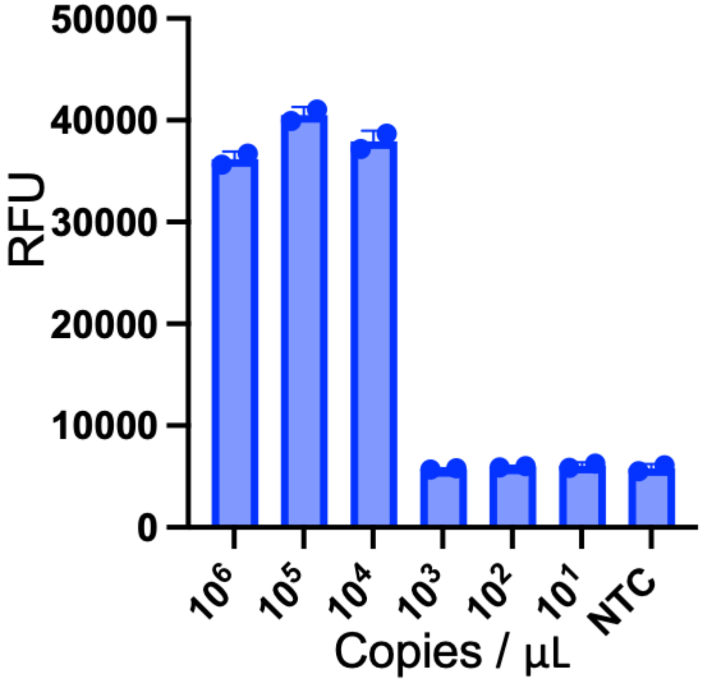

Fig. S4 | LoD using primer set A for HPV18. Primer set A was tested with synthetic dilutions of HPV18 DNA ranging from 0 to 1 million copies per µL. Plotted is the mean end-point fluorescence Of incubation in the CRISPRD reaction after two hours (n=3).

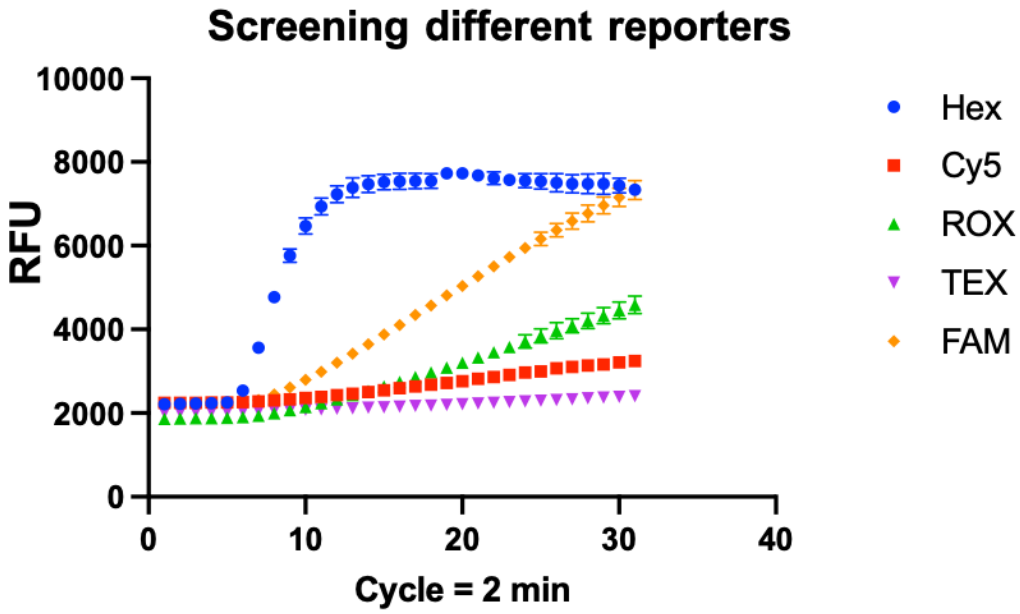

Fig. S5 | Reporter screening. The same reporter (Poly (rArG)) carrying different fluorophores was screened to check the sensitivity of each fluorophore.

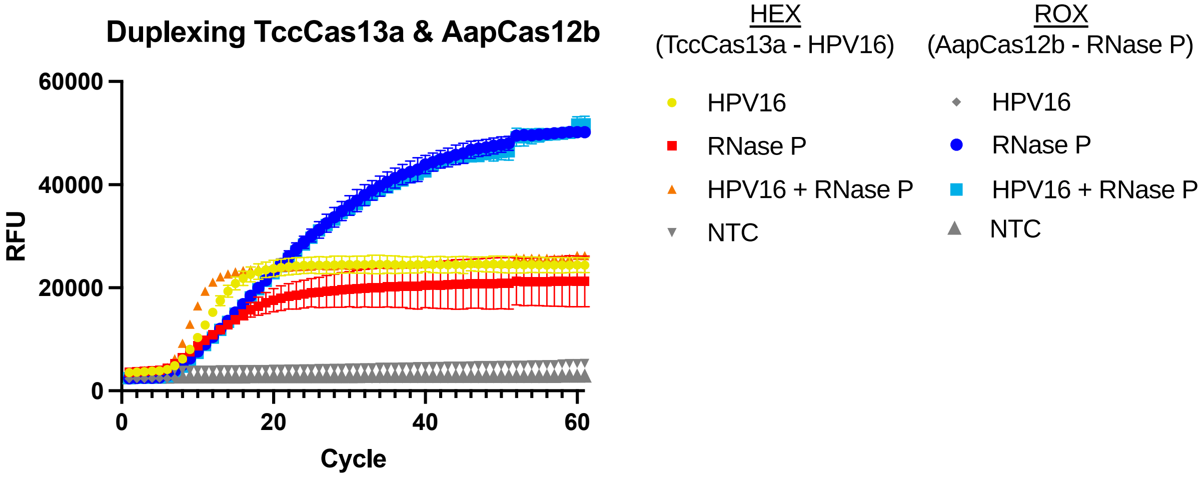

Fig. S6 | Duplexing AapCas12b with TccCas13a. A CRISPRD duplex reaction for detecting HPV16 and RNase P with TccCas13a and AapCas12b, respectively.

a

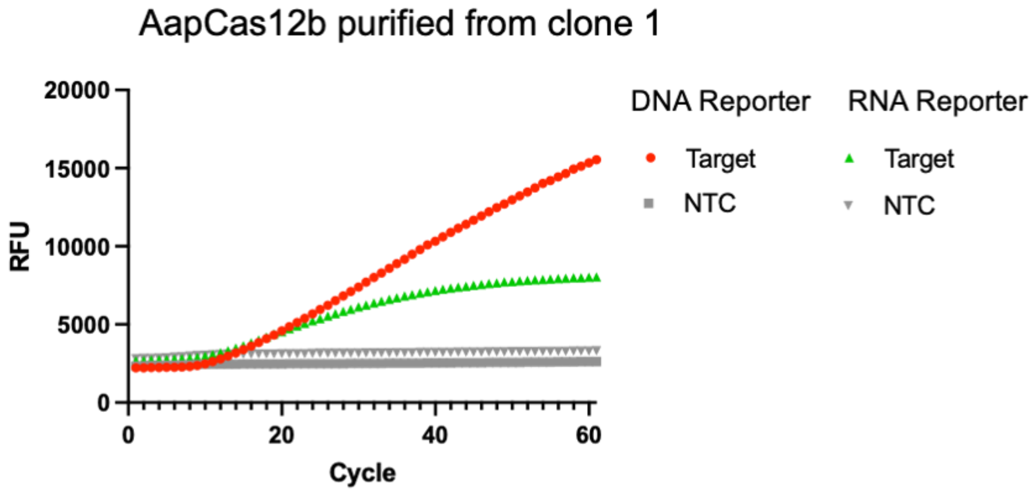

b

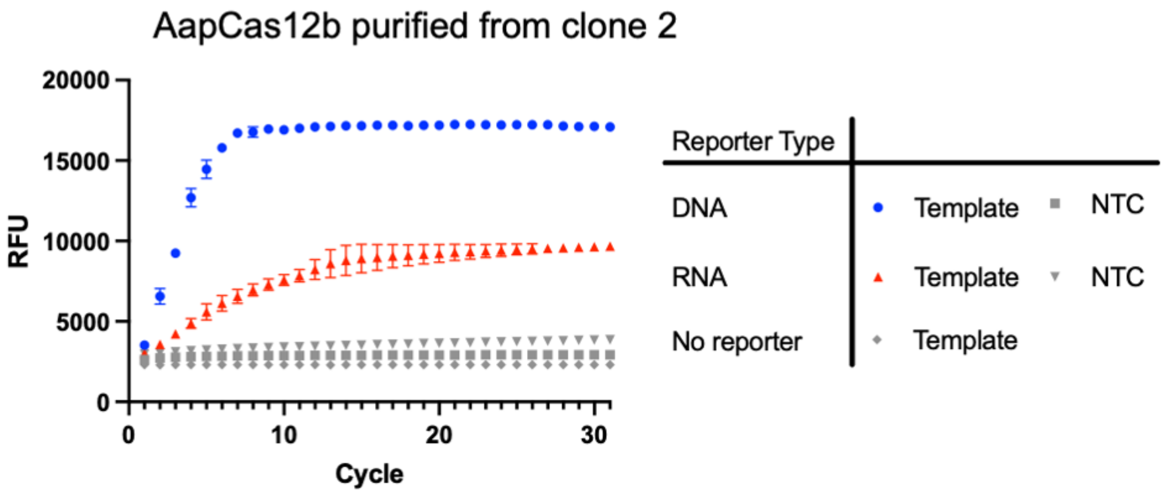

Fig. S7 | Testing the collateral cleavage activity of AapCas12b purified from two different clones. Two different clones of AapCas12b were tested to check their activity on an RNA reporter (poly (rArG)) compared to a DNA reporter (Poly T). Plotted is the mean real-time fluorescence data (n=3) and standard deviation.

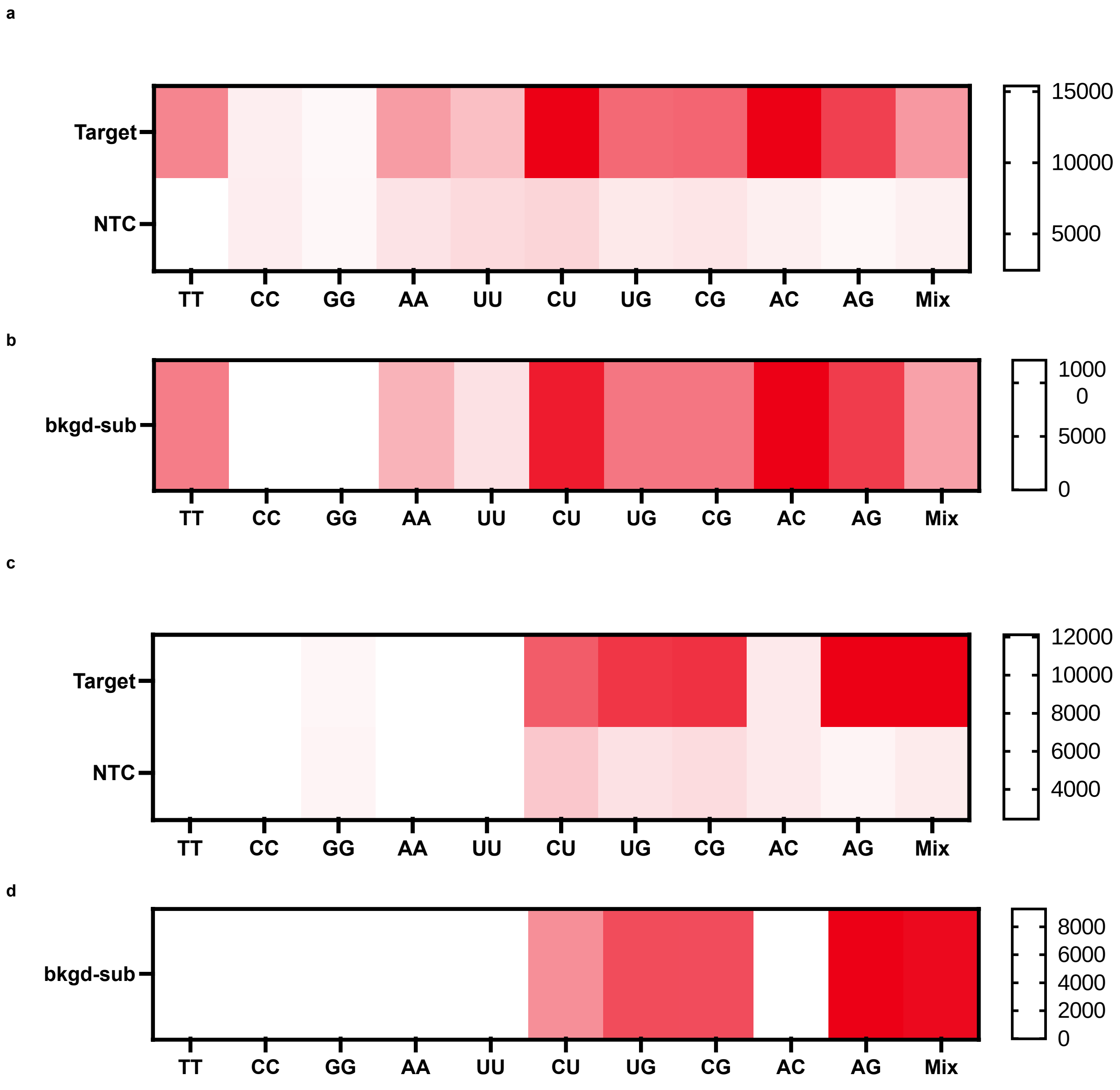

Fig. S8 | Activity of AapCas12b and TccCas13a on RNA reporters with different nucleotide compositions. Activated AapCas12b RNPs were incubated with different RNA reporters. The DNA reporter is indicated as TT; the rest are RNA reporters. Plotted is the end-point fluorescence after a 1-hour incubation (n=3). **a-b** AapCas12b activity on different reporters. **c-d** TccCas13a activity on different reporters. Target concentration is 4 ng/uL. bkgd-sub = Target fluorescence – NTC flouresence.

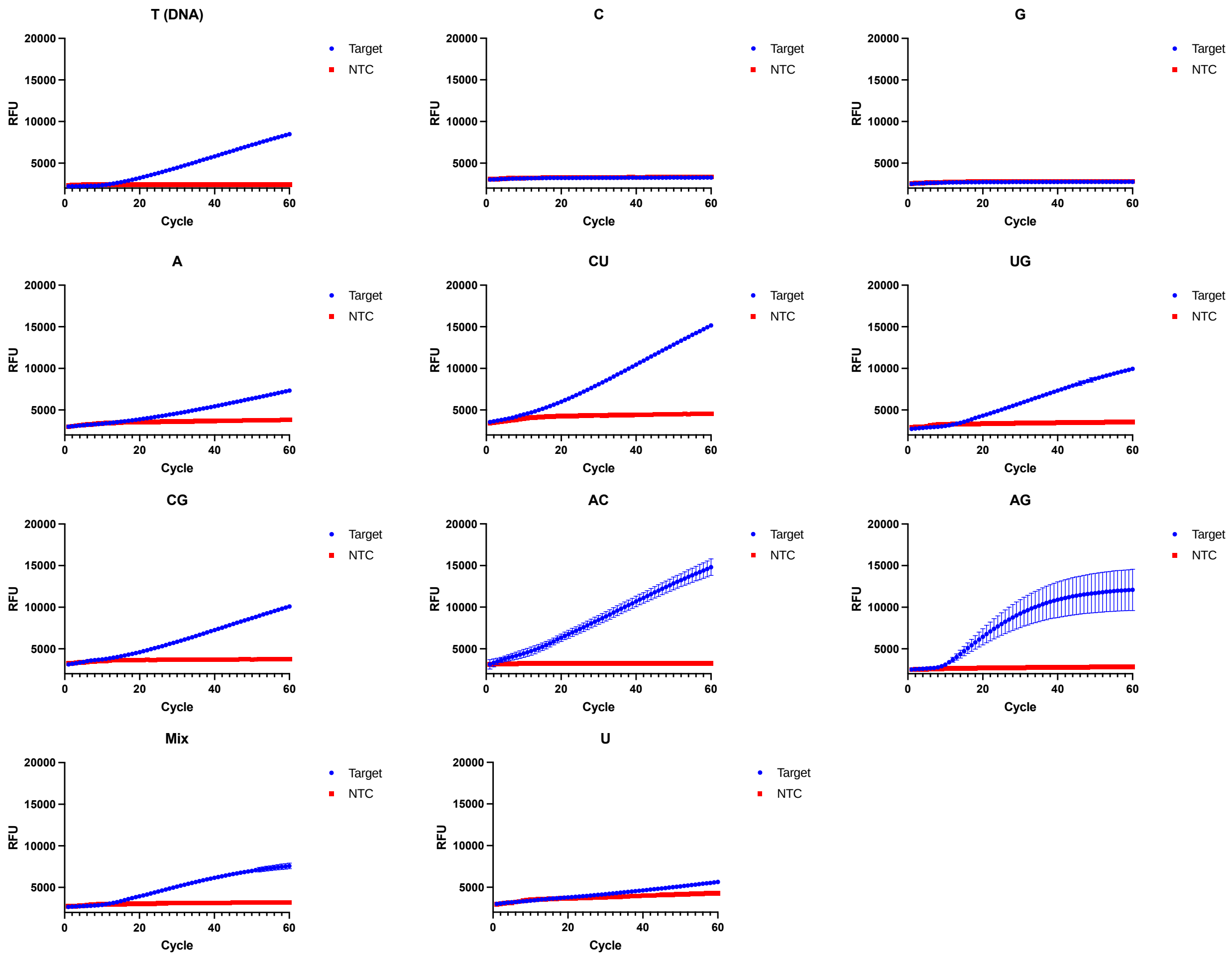

Fig. S9 | Reporter screening with AapCas12b real-time data. Activated AapCas12b RNPs were incubated with different RNA reporters. The DNA reporter is indicated as TT; the rest are RNA reporters. Plotted is the end-point fluorescence after a 1-hour incubation (n=3).

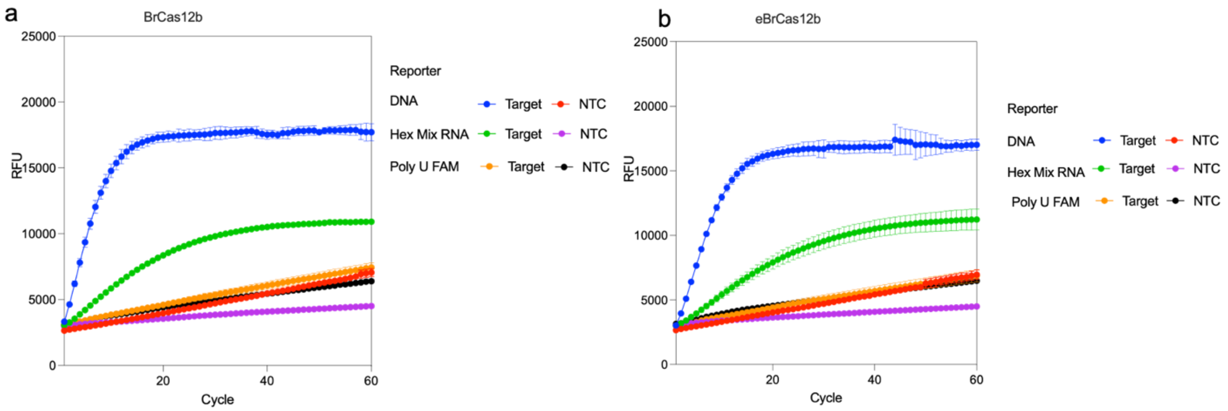

Fig. S10 | BrCas12b cleaves RNA and DNA reporters. Activated BrCas12b **(a)** and eBrCas12b **(b)** was incubated with RNA or DNA reporters.

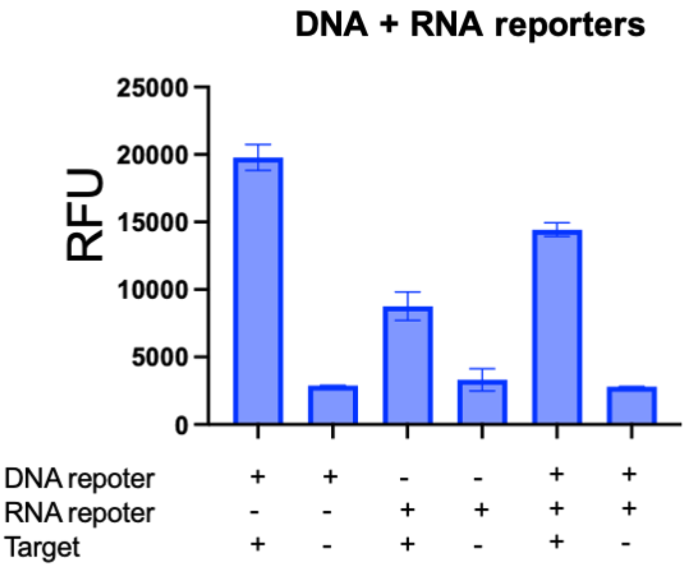

Fig. S11 | Testing AapCas12b with RNA and DNA reporters. Activated AapCas12b RNP was incubated with a DNA reporter, a RNA reporter, or both reporters in equimolar concentrations. Plotted is the end-point fluorescence (n=3).

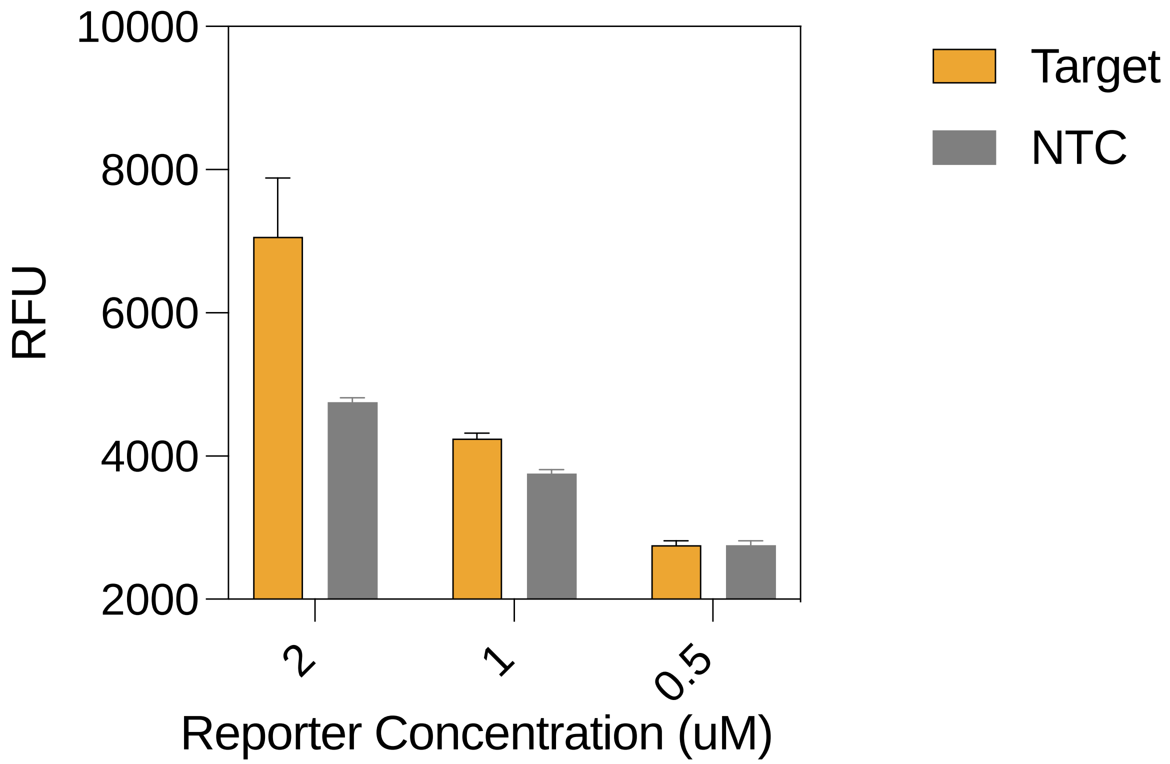

Fig. S12 | Activity of TccCas13a on Poly U RNA reporters. The CRISPRD reaction with TccCas13a RNP was incubated with RNase P (1 ng) and Fluorescence was measured after 2 hours (n=3).
